# A randomized controlled trial examining the efficacy of an internet-based cognitive behavioral therapy program for adolescents with anxiety disorders

**DOI:** 10.1371/journal.pone.0222485

**Published:** 2019-09-18

**Authors:** Silke Stjerneklar, Esben Hougaard, Lauren F. McLellan, Mikael Thastum

**Affiliations:** 1 Department of Psychology and Behavioral Sciences, School of Business and Social Sciences, Aarhus University, Aarhus, Denmark; 2 Centre for Emotional Health, Department of Psychology, Macquarie University, New South Wales, North Ryde, Australia; IRCCS E. Medea, ITALY

## Abstract

**Background:**

Anxiety disorders are highly prevalent in adolescence, but access to health care services is limited and only few receive professional help. Internet-based cognitive behavioral therapy (ICBT) has been proposed to increase accessibility and reduce costs of treatment.

**Objective:**

The study evaluated the efficacy of a Danish version of the guided ICBT program ChilledOut Online, developed at the Centre for Emotional Health, Macquarie University, Australia.

**Method:**

At the Centre for Psychological Treatment of Children and Adolescents, Aarhus University, Denmark, a randomized controlled trial was conducted with 70 adolescents (13–17 years) with anxiety disorders according to DSM-IV. Participants were randomly assigned to a 14-weeks therapist-guided ICBT or to a waitlist condition. Outcomes were evaluated post-treatment and at 3- and 12-month follow-up.

**Results:**

At post-treatment, the ICBT group significantly outperformed the waitlist condition with moderate to large between-group effect sizes on diagnostic severity and anxiety symptoms rated by clinicians, and by adolescents and their parents. Forty percent of adolescents in ICBT were free of their primary diagnosis compared to 16% in the waitlist condition. Treatment gains were maintained at 3- and 12-month follow-up.

**Conclusion:**

Results of the study provide support for the efficacy of guided ICBT for adolescents with anxiety disorders.

**Trial registration:**

ClinicalTrials.gov: NCT02535403.

## Introduction

Anxiety disorders are common in adolescence with 5–12% of youths from western countries suffering from the disorder [1–3]. When left untreated, anxiety disorders in adolescence tend to have a chronic developmental course, often predicting anxiety and depression into adulthood [4–6]. Cognitive behavioral therapy (CBT) has proven effective in treating adolescent anxiety disorders with studies showing large treatment effects in individual and group treatment formats [7]. However, access to health care services is limited and it is estimated that only around 25% of clinically anxious youth receive professional help [8–10]. Several reasons have been suggested for this, including insufficient knowledge about and limited accessibility of available mental health services, lack of trained therapists, and high therapy costs [11, 12]. Adolescents may be especially reluctant to seek help for emotional problems, even if treatment facilities are available [13–15]. Among the most frequently mentioned help-seeking barriers for adolescents are perceived social stigma [12, 16], concerns about privacy and confidentiality [12], preference for self-reliance [17, 18], and worries concerning treatment costs, transportation or waiting times [19]. Clearly, there is a need for alternative treatment delivery methods for anxiety disordered youths that strive to overcome such barriers.

Internet-based CBT (ICBT) has been proposed as a means to increase access to, and reduce costs of, psychological interventions. ICBT typically provides therapeutic content comparable to that of regular face-to-face CBT and is typically presented in modules on a weekly basis [20, 21]. ICBT interventions can be either self-directed or therapist-guided. Guidance may refer to any sort of support from a coach or a therapist, for example automated reminders, asynchronous email correspondence, brief scheduled phone calls or real-time chat [22]. Generally, research suggests that regular contact with a therapist or a coach, providing some sort of support to the user, substantially increases program usage, minimizes drop-out and improves outcome [23–25].

ICBT for adults has been widely researched with substantial evidence of effectiveness for a range of anxiety disorders [22, 26–30]. ICBT may be particularly appropriate for adolescents as it has the potential to overcome many of the barriers to seeking help for this age group, particularly less social stigma, greater anonymity, a higher degree of self-determination, reduced expenses, and eliminated travel time [27, 31–34]. Moreover, as ‘digital natives’ adolescents are highly skilled in the use of computer technologies, engaging in daily activities on the internet and commonly seeking health information online [35–38].

An emerging body of research shows promising results for the ICBT treatment of anxiety disorders in children and adolescents. Four recent meta-analyses of children, adolescents and young adults (age range 5–25) with anxiety and/or depressive symptoms [39–42] indicate that ICBT can be an effective intervention. In a systematic meta-review focusing more broadly on digital health interventions (including wearable technologies, smartphone apps, computer-assisted and internet-based therapies among others) for children, adolescents and young adults, through meta-analyses comparing the experimental condition to no treatment conditions (e.g. waitlist, placebo) Hollis et al. [43] demonstrated moderate-to-large effect sizes (ESs) on anxiety symptoms (Hedges’ *g* = 0.53–1.41). None of the above mentioned meta-analyses examined the effect of ICBT for adolescents specifically, but those that investigated age as a moderator of outcome [39–41] found better results for older youths compared to younger, although age ranges varied across studies.

When looking exclusively at ICBT with anxious adolescents, studies are sparse [44–48]. In an RCT including 19 speech-anxious high school students (aged 15–21), Tillfors et al. [44] showed that nine weeks of guided ICBT adapted from a self-help manual [49] reduced social anxiety at post-treatment with a large ES (Cohen’s *d* = 1.28) compared to a waitlist control (WL) group. Spence et al. [46] conducted an RCT with 115 clinically anxious adolescents (aged 12–18) comparing the 12-week generic ICBT program BRAVE for teenagers-ONLINE with clinic-based face-to-face CBT. At post-treatment both groups showed significant reductions in anxiety without significant differences between them. Thirty four percent were free of their primary anxiety diagnosis in the ICBT condition (intent to treat) compared with 30% in the face-to-face condition. Also, both the online group and the face-to-face group showed comparable reductions in self-rated anxiety symptoms on The Spence Children’s Anxiety Scale [SCAS; [50]] Child version post-treatment [46]. Lenhard et al. [48] conducted an RCT with 67 adolescents (aged 12–17) diagnosed with obsessive-compulsive disorder (OCD). Results showed that the 12-week clinician and parent supported ICBT-intervention was superior to WL on the clinician-administered interview Children’s Yale-Brown Obsessive-Compulsive Scale [51] with an ES of *d* = 0.69 (*p* < 0.001).

The present study investigates a Danish version of *the ChilledOut Online program* [52] developed at the Centre for Emotional Health, Macquarie University, Australia. The program builds on the structure and content of the manualized group-CBT program *Chilled* [53] for adolescents with anxiety disorders. The program has not yet been evaluated, although a prior CD-ROM version, *the Cool Teens program* [54, 55], has been demonstrated efficacious in an RCT with 43 adolescents [56]. An initial feasibility study indicated that the Danish version of ChilledOut Online could be a feasible psychological intervention for adolescents with anxiety disorders with promising results [57].

### Aim and hypotheses

The present study examined the efficacy of ChilledOut Online using a randomized waitlist-controlled design with follow-up assessments at 3- and 12-month post-intervention. We hypothesized that the ICBT intervention would demonstrate superiority to the WL condition primarily by reducing diagnostic severity and anxiety symptoms, but also by reducing symptoms of depression and improving self-efficacy and quality of life. Additionally, we expected these outcomes to be maintained at follow-ups.

## Methods

### Participants and recruitment

The study took place at the Centre for Psychological Treatment of Children and Adolescents (CEBU), a research and teaching facility at the Department of Psychology and Behavioral Sciences, Aarhus University, Denmark. The study was approved on May 7, 2015 by the local Ethics Committee of Central Denmark Region (1-10-72-98-15) and by the Danish Data Protection Agency. Furthermore, the trial was registered within ClinicalTrials.gov, registration number: NCT02535403.

Inclusion criteria were: (a) 13 to 17 years of age; (b) a primary anxiety disorder diagnosis according to the *Diagnostic and Statistical Manual of Mental Disorders* (4th ed.; DSM–IV; [58]); (c) direct access to a home computer with internet; and (d) the ability to read and write in Danish. Exclusion criteria were: (a) severe comorbid depression (Clinical Severity Rating > 5 as measured with the Anxiety Disorders Interview Schedule for DSM-IV, Child and Parent Versions (ADIS-IV C/P; [59]); (b) substance abuse; (c) current severe self-harm or suicidal ideation; (d) pervasive developmental disorder; (e) learning disorder or intellectual disability; and (f) psychotic symptoms.

Participants were recruited from May 15 to December 18, 2015 and the last follow-up assessment was obtained by March 1, 2017. Although the recruitment process was initiated prior to trial registration (August 28, 2015), baseline assessments and enrolment was not commenced until registration had been confirmed. Families had referred themselves to the CEBU secretary in response to postings on the website or recommendations from local community health services. Interested families were invited to send in a brief description of the adolescent’s major problems. If deemed eligible for the present study, families were contacted for a semi-structured diagnostic telephone interview and sent a package of online questionnaires (see full description under Measures). Those who met inclusion criteria were given verbal and written information about the study procedures. After adolescents and their parents provided written consent, they were included in the study and randomized to condition. The randomization sequence was created with an online computer random number generator using permuted block design with a fixed block size of 10 at a 1:1 allocation ratio to the ICBT or the WL condition. The sequence list was kept concealed from researchers and therapists, stored by an external secretary at the University who administered group assignment to included participants according to the randomization sequence. Participants in both conditions were encouraged not to engage in other forms of treatment nor make changes to their use of psychiatric medication during the acute treatment and waitlist period.

### Procedures

Adolescents randomized to the WL condition were instructed to wait 14 weeks for treatment. During this period, participants did not have any form of planned contact with the project team. After 14 weeks, adolescents and parents recompleted the assessment measures, took part in a second telephone interview, and were offered the ICBT treatment. Adolescents randomized to the ICBT condition were informed of their allocation over the phone by their appointed therapist and provided introductory information about the program. During this 20-minute introductory phone call, the course of the intervention was summarized, adolescents were given a brief introduction to the program website and functions, and therapists and adolescents agreed upon the time and dates of their weekly supportive phone calls. The adolescent then received a letter outlining the treatment start date and program information, i.e. the program URL, a personal username, a temporary password, the therapist e-mail address, a photo of the therapist, and a calendar plan of the future therapist phone calls. All therapist calls were recorded using Crystal Gears® Ver. 2.00 RTM for supervision purposes and to ensure integrity of the intervention.

Adolescents’ diagnostic status was assessed at baseline (pre), after the intervention (post), and at three-month follow-up with the ADIS-IV C/P [59] administered via telephone. The interviews were conducted by trained graduate psychology students working at the Center, closely supervised by clinical psychologists, experienced in using the ADIS-IV. Assessors were blind to group allocation at pre-assessment and of participants’ prior diagnoses at post and follow-up. Assessors were also blind to group allocation at post assessment, although most families did reveal their allocation status during the post interview. All interviews were recorded using Crystal Gears® Ver. 2.00 RTM. One of two trained assessors re-assessed 14 (20%) of the audio-recorded baseline interviews to allow for subsequent evaluation of interrater reliability. Adolescents and both parents separately completed online self-report questionnaires at pre, post, three- and twelve-month follow-up. Assessment at 12-month follow-up only included SCAS, CALIS and WHO-5 (see reference to the scales below). The questionnaires were translated into Danish using the forward-backward translation process and administered through an electronic data collection platform, SurveyXact. Families failing to complete the questionnaires at any assessment point received three weekly e-mail reminders followed by a phone call after four weeks from their appointed therapist.

### Measures

#### Primary outcome measures

**Clinician-rated diagnostic severity.** The ADIS-IV C/P [59] is a semi-structured diagnostic interview conducted with the adolescent and one parent separately to assess the diagnostic criteria of anxiety disorders in accordance with DSM-IV [58]. In addition to anxiety disorders, the ADIS-IV permits assessment of other disorders often associated with anxiety, including dysthymia, depression, ADHD, oppositional defiant disorder, and conduct disorder. The severity of each diagnosis is evaluated by a clinician on a nine-point Likert scale (0 = not at all disturbing; 8 = severely disturbing), the Clinical Severity Rating (CSR), using information from both adolescent and parent. Disorders were considered clinically impairing if clinicians provided a CSR of 4 or greater. When more than one disorder was present, clinicians identified the primary diagnosis (defined as the most impairing disorder). The ADIS-IV has shown good to excellent test-retest reliability for the presence of specific diagnoses and CSR [60]. Concurrent validity of the anxiety disorders section has also been established [61]. Administering the ADIS-IV over the telephone has also yielded high inter-rater reliability and validity, comparable to face-to-face administration [62]. The inter-rater reliability (Cohen’s Kappa) for the primary anxiety diagnoses in this study was excellent, *κ* = 0.80 (95% CI: 0.556–1.038; *p* < 0.001). The intraclass correlation coefficient (ICC; two-way random effects model, consistency, individual raters), was fair for the CSR of the primary anxiety diagnosis (CSR_prim_), ICC = 0.419 (95% CI: -0.121–0.768; *p* = 0.060) and good for the CSR of all anxiety diagnoses (CSR_all_), ICC = 0.731 (95% CI: 0.348–0.905; *p* = 0.001).

**Adolescent- and parent-rated anxiety symptoms.** The Spence Children’s Anxiety Scale (SCAS; [50]) measures self-rated anxiety symptoms in six domains: social phobia (SoP), generalized anxiety disorder (GAD), specific phobia (SP; ‘fear of physical injury’), separation anxiety disorder (SAD), OCD, and panic disorder (PD) with agoraphobia (AP). However, in the present study only the total score measure of overall symptom severity was used. The questionnaire contains 38 items, each rated on a four-point Likert scale from zero to three. Higher scores indicate higher anxiety symptom levels. The scale is filled out by the adolescent (SCAS-C) and by the each of the parents (SCAS-P). The SCAS-C includes six additional positive filler items. The Danish version of the SCAS has demonstrated good to excellent internal consistency for the total score in a clinical, as well as a community sample, and good test-retest reliability in a community sample [63]. In the present study, internal consistency (Cronbach’s *α*) for the total score was good to excellent for adolescent (*α* = 0.88), mother (*α* = 0.89), and father (*α* = 0.90) report versions of the scale.

#### Secondary measures

**Anxiety life interference.** The Child Anxiety Life Inference Scale (CALIS; [64]) measures the interference of youth anxiety on various areas of life functioning including family, friends, school, and extracurricular activities. Items are evaluated separately by the adolescent (nine items) and their parents (nine items) on five-point Likert scales (0 = not at all; 4 = a great deal). Parents also rate the interference of the adolescents’ anxiety on their own life including their relationship with spouse, friends, family, career choices and general level of stress (seven items). For the present study, only the total scores on parent reports, i.e. the sum of the rated interference on child and parent life (16 items), was used. The CALIS has demonstrated satisfactory internal consistency and moderate retest reliability in its original English language version [64]. Internal consistency in the current sample (Cronbach’s *α*) was acceptable for adolescent reports (*α* = 0.77), and good for mother and father reports (*α* = 0.87 and *α* = 0.89, respectively).

**Depressive symptoms.** The short version of the Moods and Feelings Questionnaire (S-MFQ; [65]) measures youth depressive symptoms within the last two weeks as evaluated separately by adolescents and parents. The questionnaire consists of 13 items rated on a three-point Likert scale (0 = not true; 2 = true). The English version of the S-MFQ has shown good internal consistency on both youth self-report and parent-report in [65]. In the present study, internal consistency (Cronbach’s *α*) was excellent for adolescent report (*α* = 0.90), and good for mother and father reports (*α* = 0.88 and *α* = 0.87, respectively).

**Self-efficacy.** The Self-Efficacy Questionnaire for Children (SEQ-C; [66]) is a 24-item measure of self-efficacy assessing youth perceptions of personal strengths and competencies in three domains: academic (ability to succeed in school and display appropriate learning behaviors), social (ability to get along with and relate to peers), and emotional (ability to regulate negative emotions). Items are rated on a five-point Likert scale (1 = not at all; 5 = very well). In its original English version, the SEQ-C has shown high internal reliability [66], good internal consistency [67], and criterion validity has been established [68]. In the current study, internal consistency (Cronbach’s *α*) for the total scale was good, *α* = 0.89.

**Mental well-being.** The WHO-5 Well-being Index Questionnaire (WHO-5; [69]) is a five-item self-report measure of respondents subjective mental well-being during the previous two weeks covering positive mood, vitality and general interest. Each item is rated on a six-point Likert scale (0 = not present; 5 = constantly present). Raw scores range from zero to 25. When interpreting changes in WHO-5 scores, the scale is translated into 0–100%, and the percentage value is used, calculated by multiplying raw scores by four. By convention, a score between 100 and 50 indicate an acceptable life-quality, a score between 49 and 30 indicate a moderately reduced life-quality, while a score ≤ 30 indicate a pathologically reduced well-being, i.e. moderate depression. The English version of the scale has demonstrated excellent internal consistency and good criterion validity [70, 71]. Internal consistency (Cronbach’s *α*) in the present sample was good, *α* = 0.86.

**Computer experience.** At pre-treatment, adolescents were asked to rate their computer experience by answering the question: ‘How comfortable do you feel using the computer and the internet?’ rated on a four-point Likert scale (1 = not comfortable at all; 4 = very comfortable).

**Treatment satisfaction.** Satisfaction with the ICBT intervention was measured in adolescents and parents post treatment using a short questionnaire adapted from The Experience of Service Questionnaire [72, 73]. Separate versions were used for adolescents (seven items) and parents (nine items), rating affirmative statements on a three-point Likert scale (1 = not true; 2 = partly true; 3 = true), e.g. ‘The treatment helped me/my child’, ‘We feel better in the family now compared to before the treatment’, and ‘If a friend needed this type of help, I would recommend him/her to contact the Centre’. The questionnaire ended with an open section inviting respondents to provide any feedback in relation to the intervention.

**Program activity and support.** Adolescents’ program activity was automatically registered at the website server as number of logins, number of module visits and number of module components activated. Therapists kept a record of all contact with adolescents and parents, noting the duration of each phone call and all e-mail correspondence throughout the intervention. Moreover, adolescents were asked to rate their average time spent per week on program relevant activities (on- and offline separately) at post treatment, and parents were asked to report their average time spent per week helping their child with program relevant activities (on- and off-line combined).

The following measures were administered but not reported here: *The Strengths and Difficulties Questionnaire* [74, 75]; *The Working Alliance Inventory-Short Form* [76].

#### Treatment

ChilledOut Online is a treatment program based on the Cool Kids and Chilled anxiety management program developed at Macquarie University, Sydney, Australia [52]. The program teaches CBT strategies for adolescents through eight online modules of approximately 30 minutes, with a focus on psychoeducation, cognitive restructuring and graded exposure. To allow for flexibility and personal learning preferences, adolescents were able to access all modules at treatment start. To guide progress through the program, adolescents were however encouraged to complete all eight modules (and module content) in the order they appeared within the 14-week intervention period, after which they would have access to the web site for another three months. Program content such as goal setting, realistic thinking, problem solving, and assertiveness, is presented through a combination of multimedia formats, i.e. text, audio, illustrations, cartoons, worksheets, and video vignettes. An overview of module content is presented in Table 1. The program includes six video cases of adolescents with different anxiety issues, who reappear throughout the program as they demonstrate various program skills to manage their problems. Furthermore, each module contains homework practice tasks that adolescents are encouraged to complete. Once a week, participants are asked to rate nine statements concerning the interference of anxiety in the following areas of their lives: (1) getting along with parents, (2) getting along with siblings, (3) spending time with friends outside school, (4) finishing homework assignments, (5) spending time with classmates in recess, (6) play sports, (7) attending leisure activities, (8) completing daily routines, and (9) general mood. The statements enable participants to view a progress chart of their weekly total scores and thus track changes during the intervention. For the present study, the original Australian version of ChilledOut Online [52] was translated and revised according to Danish language. Revisions primarily involved recording of new video material with Danish characters; all other translations were as close to the original program as possible.

**Table 1:** Module content and homework practice tasks.

Module title	Module content	Homework practice tasks
1. Understanding anxiety	• How to use the program • Psychoeducation about anxiety	• Complete an anxiety self-assessment questionnaire to get overview of present anxiety issues
2. Setting goals	• Learning to set goals • Rewards • Measuring feelings on a worry scale	• Identify goals • Make a list of possible rewards
3. Realistic thinking I	• Linking thoughts and feelings • Negative thinking • Identifying and challenging unrealistic thoughts (cognitive restructuring)	• Practice realistic thinking • Rewards
4. Stepladders I	• Identifying and categorizing fears and worries • Graded exposure using stepladders	• Create the first stepladder • Plan the execution of the stepladder • Practice steps until goal is reached
5. Stepladders II	• Revising old and creating new stepladders • Behavioral experiments • Help solving stepladder barriers and difficulties	• Create more stepladders • Practice behavioral experiments
6. Realistic thinking II	• Simplifying realistic thinking (in my mind) • Acting as if • Surfing emotions	• Make list of useful questions and thoughts for realistic thinking • Continue working on stepladders
7. Other coping skills	• Problem solving • Constructive feedback • Assertive communication • Calming activities	• Practice problem solving and assertiveness
8. Staying chilled	• Skills overview and maintenance • Relapse prevention	• Continue to practice skills

*Note*. Table originally published in Stjerneklar et al. [57]

Adolescents received a weekly phone call from their therapist set to a duration of approximately 20 minutes, with a focus on assisting learning of program skills and strategies, offering technical assistance, giving feedback about homework tasks, and encouraging program use during times of low motivation. The therapists were graduate psychology students in a clinical training course supervised by psychologists working at CEBU. All therapists received training in cognitive behavioral therapy and a thorough introduction to the program. Moreover, all therapists were equipped with a semi-structured “manual” with topics to be dealt with in the calls. The therapists had access to participants’ module activities and worksheets throughout the program. At three-month follow-up, adolescents received a booster phone call from their therapist with the aim of consolidating previously learned skills.

Shortly before treatment start, parents received *the ChilledOut Parent Companion* describing their role in treatment and providing them with an explanation of the program’s core treatment strategies (psychoeducation, goal setting, cognitive restructuring, and graded exposure). Furthermore, the handout included advice on how to support their teenager in his/her efforts to overcome anxiety. Parents are encouraged to lend support and motivational encouragement to their child during treatment. However, they are also advised to be sensitive to their child’s need for autonomy. Within the first two weeks of the intervention, parents received a brief phone call in which the therapist introduced him- or her-self and answered any questions about the program. During the remaining intervention period, parents were invited to contact the therapist through e-mail and, if needed, they were offered a short phone call from the therapist. Based on parent feedback from a previous feasibility trial of the ChilledOut Online program [57], parents were invited to sign up for a closed online network to facilitate group exchanges and support.

### Statistical analyses

Allowing for approximately 20% dropout (cf. [77]), the study was acceptably powered to detect a large ES (Cohen’s *d* = 0.80) with a power of .80 and two-tailed *α* = .05 [78].

Baseline group differences were explored using independent samples *t*-tests, *χ*^2^-tests or Fisher’s exact test (two-sided) as appropriate. Pre–post comparisons between the two conditions were analyzed as (1) number of participants free of primary or all anxiety diagnoses at post-treatment; (2) degree of change on continuous outcome measures from pre to post; and (3) number of participants with clinically significant change on the SCAS.

Mixed linear models (MLMs) were used to compare the two conditions from pre to post on continuous outcome variables. MLMs tolerate missing values and thus do not unnecessarily compromise statistical power. All MLMs were based on the intention-to-treat sample (N = 70) without any ad hoc imputations of missing values, which is recommended over other procedures of handling missing data in longitudinal clinical trials [79]. All MLMs were estimated with the full maximum likelihood method. Data were hierarchically arranged in two levels, with time at level 1 nested within individuals at level 2. For the MLMs of the pre-post comparisons, fixed effects were specified for time (pre and post), group (ICBT and WL), and the time × group interaction. Models included a random intercept. With three dependent variables on SCAS, CALIS and S-MFQ (adolescents, mothers and fathers respectively), for these measures we employed a family-wise Bonferroni-corrected significance level of *p* = 0.017 (0.05/3) for each set of analyses.

Using Jacobson and Truax’s [80] method, the reliable change index (RCI) and clinical cut-off score were determined on SCAS. The formula used was: $RCI=1.96\times (\sqrt{2}\times {\left(SD\times \sqrt{1-r_{xx}}\right)}^{2})$, where SD is the standard deviation of the standardized sample and *r*_*xx*_ is the reliability of the measure (internal consistency, Cronbach’s *α*). The RCI was calculated using standard deviations and internal consistency ratings from Danish norm data [63]. The clinical cut-off score (CS_cut-off_) defined as the mid-point between the clinical and non-clinical population, was calculated with the formula: CS_cut−off_ = ((M_clin_×SD_norm_)+(M_norm_×SD_clin_))÷(SD_norm_+SD_clin_). The CS_cut-off_ was calculated from Danish community and clinical norms split into gender and age groups reported in Arendt et al. [63]. As suggested by Jacobson and Truax [80], participants achieving statistically reliable change according to the RCI were considered improved, whereas those achieving both statistically reliable and clinical change were considered recovered. Calculations of improvement and recovery were based on participants who completed questionnaires.

Only participants randomized to the ICBT condition were included in the follow-up analyses. Analyses of treatment outcome maintenance were conducted on SCAS, CALIS and WHO-5 from pre to 3- and 12-month follow-up and for ADIS CSR from pre to 3-month follow-up using MLMs. For models including more than two measuring points (e.g., analyses of treatment maintenance from pre to 3- or 12-month follow-up), the slope was specified as random if it significantly improved model fit as evaluated by a change in the –2LL fit statistics [81].

For all analyses ES calculations were based on observed values and expressed as Cohen’s *d*, with 0.2, 0.5, and 0.8 considered a small, medium, and large ES, respectively [78]. For the between group analyses, Cohen’s *d* was derived by the following formula: *d* = (M_diffICBT_÷SD_ICBTpooled_)−(M_diffWL_÷SD_WLpooled_), were M_diff ICBT_ and M_diff WL_ are the means of the pre-post difference scores for the ICBT and WL group respectively, and SD_ICBT pooled_ and SD_WL pooled_ are the pooled standard deviations of the two groups, calculated by the formula: ${SD}_{pooled}=\surd (({SD}_{pre}^{2}+{SD}_{post}^{2})÷2)$. Within-group ES’s were calculated by dividing the difference between the pre- and post-assessment means with the pooled standard deviation of those two means: *d* = (M_pre_−M_post_)÷SD_pooled_.

Study dropouts were defined as participants who had not remained in contact with the therapist for the entire 14 weeks of intervention. Otherwise participants were considered completers, regardless of their number of completed modules or amount of completed therapist calls.

All statistical analyses were carried out using IBM® SPSS® statistics, v.24.0 (Armonk, NY: IBM Corp.).

## Results

### Study flow and baseline participant characteristics

**Fig 1:**
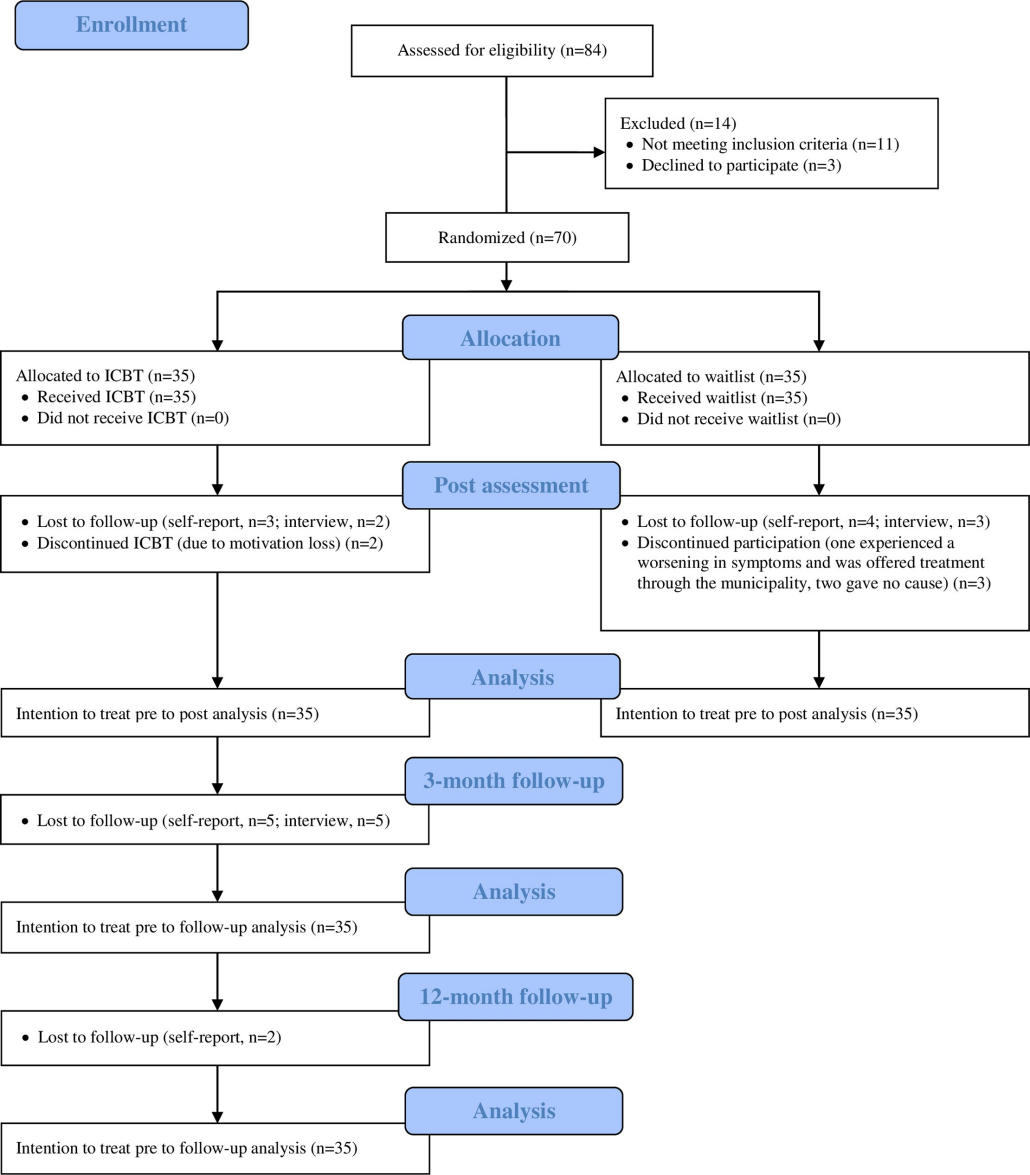
Study flow-chart.

A total of 120 adolescents were recruited. Eighty-four adolescents presented themselves with eligible problems and thus received questionnaires and phone interviews (see study flow-chart, Fig 1). Fourteen adolescents were excluded, 11 of which did not meet the inclusion criteria and three declined to participate despite their initial interest. Seventy adolescents aged 13–17 (M = 15.0, SD = 1.30) met inclusion criteria and were randomly assigned to ICBT (n = 35, 83% females) or WL (n = 35, 73% females).

Five participants (7%) dropped out prior to post-assessments. Two participants (ICBT) dropped out due to loss of motivation, one (WL) experienced a worsening in symptoms during the waitlist period (he was offered treatment through the municipality), and two (WL) gave no explanation. No significant differences were found on primary outcome pre-treatment scores (CSR and SCAS-C/P), gender, age, number of anxiety diagnoses, age at anxiety onset, previous therapeutic treatment, primary diagnosis or computer experience between dropouts and completers. Ninety-six percent (63/65) of the adolescent study completers returned the self-report questionnaire at post, while 100% (65/65) took part in the diagnostic post-interview. At 3-month follow-up, 82% (27/33) of the adolescent study completers (ICBT condition only) returned the self-report questionnaire, while 85% (28/33) completed the diagnostic interview. At 12-month follow-up, 76% (25/33) of the adolescent study completers returned the self-report questionnaire. Reasons for non-completion of questionnaires and interviews are largely unknown, as most of these participants could not be reached.

Baseline characteristics are presented in Table 2. Fifty (71%) adolescents lived with both of their parents. The sample was relatively high in socioeconomic status, with 46% households having an annual income ≥ 100.000 EUR (the average personal income in Denmark was 41.363 EUR in 2015; [82]). In terms of primary diagnosis, 28 (40%) had SoP, 11 (16%) had GAD, six (9%) had SP, eight (11%) had OCD, three (4%) had PD, nine (13%) had SAD, two (3%) had PD with AP, and three (4%) had AP without PD. Fifty-three (76%) met the diagnostic criteria for a comorbid diagnosis, 51 (73%) were diagnosed with a comorbid anxiety disorder, and six (9%) with a mood disorder. The mean number of anxiety diagnoses per adolescent at pre-treatment was 2.11 (SD = 0.93). No significant between-group differences were found for any of the primary outcome measures at pre-treatment. Likewise, no significant differences were found for any of the demographic variables except annual household income, *t* (68) = 3.345, *p* = 0.001., where families in the WL condition had higher income than families in the ICBT condition.

**Table 2:** Sample characteristics.

	ICBT (n = 35)	Waitlist (n = 35)	Total (N = 70)
Females (%)	29 (83)	26 (74)	55 (79)
Adolescent age, M (SD)	15.22 (1.32)	14.83 (1.28)	15.03 (1.30)
Living with both parents (%)	23 (66)	27 (77)	50 (71)
Parent age, mothers/fathers, M (SD)	45.86/48.84 (4.47/5.92)	46.31/47.09 (4.50/4.64)	46.08/47.95 (4.46/5.34)
Level of education of mothers/fathers (%)			
University / Doctoral degree	7/8 (20/23)	4/9 (11/26)	11/17 (16/24)
Vocational / College	26/23 (74/66)	31/19 (89/54)	57/42 (81/60)
Primary / High school	2/0 (6/0)	0/3 (0/9)	2/3 (3/4)
NA	0/4 (0/11)	0/4 (0/11)	0/8 (0/11)
Annual household income in Euro, M (SD)	79566.99 (33885.81)	108829.05 (39124.18)	94198.02 (39207.54)
>150.000 (%)	0 (0)	6 (17)	6 (9)
100.000–149.999 (%)	12 (34)	14 (40)	26 (37)
50.000–99.999 (%)	15 (43)	14 (40)	29 (41)
<50.000 (%)	8 (23)	1 (3)	9 (13)
Age of anxiety onset^a^, M (SD)	8.6 (4.16)	9.2 (3.47)	8.9 (3.81)
Primary diagnoses (%)			
Social phobia	14 (40)	14 (40)	28 (40)
Generalized anxiety disorder	6 (17)	5 (14)	11 (16)
Specific phobia	4 (11)	2 (6)	6 (9)
Obsessive-compulsive disorder	3 (9)	5 (14)	8 (11)
Separation anxiety disorder	2 (6)	7 (20)	9 (13)
Panic disorder	3 (9)	0 (0)	3 (4)
Panic disorder with AP	1 (3)	1 (3)	2 (3)
AP without panic disorder	2 (6)	1 (3)	3 (4)
Comorbid diagnoses (%)			
Anxiety disorders	29 (83)	22 (63)	51 (73)
Mood disorders	3 (9)	3 (9)	6 (9)
Externalizing disorders	0 (0)	0 (0)	0 (0)
No comorbidity	5 (14)	12 (34)	17 (24)
Number of anxiety diagnoses per adolescent, M (SD)	2.29 (0.93)	1.94 (0.91)	2.11 (0.93)
On psychopharmacological medication (%)	5 (14)	2 (6)	7 (10)
Previous therapeutic treatment (%)	21 (60)	22 (63)	43 (61)

*Note*. AP: Agoraphobia

^a^ Age of onset is based on the mean age from mother and father reports.

Regarding computer experience, thirty-three (94%) of the adolescents randomized to the ICBT condition reported feeling either ‘fairly confident’ or ‘very confident’ using computer and internet. Two (6%) reported feeling only ‘a little confident’ and none reported ‘not at all confident’.

### Program activity and support

Participants (ICBT group minus drop-outs, n = 33) completed a mean of 5.4 (SD = 2.37, range 0–8) of the eight modules available. Modules were registered as completed if participants had activated ≥ 80% of core module components (i.e., video instructions, examples and practice tasks excluding downloads and worksheets). According to website server logs, participants activated a mean of 74% (SD = 23.17, range 32–100%) of all module components. Total number of logins to the program web site ranged from seven to 51 (M = 24.4, SD = 10.57). Table 3 presents an overview of participants’ program activity and amount of therapeutic support. Ten participants (30%) completed all eight modules, three participants (9%) completed seven modules, five (15%) completed six modules, three (9%) completed five modules, two (6%) completed four modules, six (18%) completed three modules, three (9%) completed two modules, and no participants completed only one module. One participant (3%) completed zero modules according to the definition of ≥ 80% module component activation. As participants were only advised to complete modules in the numerical order they appeared, but allowed to access them in the order they preferred, approximately one third (27%) completed modules in a random order (e.g., module 1, 6 and 7), while the remaining completed them as intended. Participants received a mean of 11.0 phone calls from the therapist (SD = 1.93, range 5–14) during the intervention period, with an average call duration of 19.5 minutes (SD = 8.80, range 2–56). Parents received a mean of 1.3 therapist phone calls (SD = 0.64, range 0–3) with an average call duration of 24.0 minutes (SD = 10.62, range 3–45). Twenty-two mothers (63%) and six fathers (17%) joined the online parent network during the study period. The majority of postings and responses in the community originated from four mothers, accounting for 76% of the online activity.

**Table 3:** Program activity and support for the ICBT condition, completer sample (n = 33); mean (SD) {range}.

# modules completed	# components activated^a^	# log ins	# calls T/A	Mean duration^a^ of calls T/A	# emails T/A	# calls T/P	Mean duration^b^ of calls T/P	# emails T/P
5.4 (2.37) {0–8}	74.4 (23.17) {32.3–100}	24.4 (10.57) {7–51}	11.0 (1.93) {5–14}	19.5 (8.80) {2–56}	0.2 (0.65) {0–3}	1.3 (0.64) {0–3}	24.0 (10.62) {3–45}	0.6 (1.23) {0–6}

*Note*. #: number; T/A: between therapist and adolescent; T/P: between therapist and parent.

^a^ Components activated is reported as percentages per module

^b^ Duration is reported in minutes

### Primary outcomes

At post-treatment, of those with diagnostic post-interview data significantly more adolescents in the ICBT condition (40%) were free of their primary diagnosis, compared with adolescents in the WL condition (16%), *χ*^2^(1) = 4,89, p = .027 (Table 4 shows frequency of improvement and recovery for the intention-to-treat sample). Based on the odds ratio, the odds of a participant being free of their primary diagnosis were 3.60 times higher if they received ICBT than if they were on WL. Also, significantly more adolescents in the ICBT condition (29%) were free of all anxiety diagnoses at post, compared with adolescents in the WL condition (3%), *χ*^2^(1) = 7,89, p = .005. The odds of a participant being free of all anxiety diagnoses were 12.38 times higher if they received ICBT than if they were on WL.

**Table 4:** Frequency of improvement and recovery for the intention-to-treat sample; n (%).

	Post-treatment	3-month f/up	12-month f/up
Free of primary diagnosis			
ICBT	14/35 (40%)	12/33 (36%)	- -
WL	5/32 (16%)		
Free of all anxiety diagnoses			
ICBT	10/35 (29%)	10/33 (30%)	- -
WL	1/32 (3%)		
Improved (SCAS-C_adol_)			
ICBT	22/32 (69%)	18/27 (67%)	12/25 (48%)
WL	8/31 (26%)		
Recovered (SCAS-C_adol_)			
ICBT	14/32 (44%)	14/27 (52%)	10/25 (40%)
WL	2/31 (6%)		
Improved (SCAS-P_mother_)			
ICBT	24/35 (69%)	23/33 (70%)	20/31 (65%)
WL	7/32 (22%)		
Recovered (SCAS-P_mother_)			
ICBT	9/35 (26%)	13/33 (39%)	14/31 (45%)
WL	2/32 (6%)		
Improved (SCAS-P_father_)			
ICBT	9/25 (35%)	14/23 (61%)	14/21 (67%)
WL	5/27 (19%)		
Recovered (SCAS-P_father_)			
ICBT	1/25 (4%)	7/23 (30%)	9/21 (43%)
WL	2/27 (7%)		

Note. SCAS-C/P: Spence Children’s Anxiety Scale Child/Parent version. f/up: follow-up.

Table 5 shows mean scores, standard deviations, ES’s and *p*-values for all outcome scales at pre and post for the ICBT and WL condition and at follow-up for the ICBT condition. The number of adolescents in the ICBT group free of their primary diagnosis at post-treatment (intention-to-treat sample) was distributed as follows: SAD = 0% (0/2), SoP = 21% (3/14), SP = 50% (2/4), PD = 67% (2/3), PD with AP = 0% (0/1), AP without PD = 0% (0/2), GAD = 83% (5/6), OCD = 67% (2/3). Although numerically diverse, a Fisher’s Exact test with eight groups (all diagnoses) revealed a non-significant association (*p* = .062) between type of primary anxiety diagnosis pre-treatment and whether or not the adolescents were free of these post-treatment (Fisher’s Exact test value = 11.05).

**Table 5:** Means, standard deviations and effect sizes of primary and secondary outcomes.

	Unadjusted mean (standard deviation) {valid n}				Effect size; *p*-value				
	Pre-treatment	Post-treatment	3-month f/up	12-month f/up	Within-group	Within-group	Between-group	Within-group	Within-group
					pre to post	pre to f/up	ICBT vs. WL	post to 3 f/up	3 to 12 f/up
ADIS CSR_prim_									
ICBT	6.69 (0.68) {35}	3.83 (2.65) {35}	3.88 (2.56) {33}		*d* = 1.48, *p* < .001	*d* = 1.50, *p* < .001^a^	*d* = .65, *p* = .022	*d* = -.02, *p* = .495	
WL	6.54 (0.92) {35}	5.09 (2.29) {32}			*d* = .83, *p* = .001				
ADIS CSR_all_									
ICBT	13.17 (5.28) {35}	6.89 (4.56) {35}	6.39 (4.64) {33}		*d* = 1.27, *p* < .001	*d* = 1.36, *p* < .001^a^	*d* = .83, *p* = .002	*d* = .11, *p* = .482	
WL	11.17 (4.42) {35}	9.28 (4.13) {32}			*d* = .44, *p* = .012				
SCAS-C_adol_									
ICBT	47.29 (15.38) {35}	31.88 (16.06) {32}	28.74 (16.08) {27}	33.36 (12.64) {25}	*d* = .98, *p* < .001	*d* = .99, *p* < .001^b^	*d* = .68, *p* < .001	*d* = .20, *p* = .054	*d* = -.32, *p* = .056
WL	45.60 (15.79) {35}	40.19 (19.90) {31}			*d* = .30, *p* = .038				
SCAS-P_mother_									
ICBT	48.23 (17.28) {35}	29.43 (13.60) {35}	28.73 (16.57) {33}	28.58 (18.58) {31}	*d* = 1.21, *p* < .001	*d* = 1.10, *p* < .001^b^	*d* = 1.12, *p* < .001	*d* = .05, *p* = .548	*d* = .01, *p* = .849
WL	42.71 (14.85) {35}	41.31 (17.54) {32}			*d* = .09, *p* = .632				
SCAS-P_father_									
ICBT	41.45 (19.18) {31}	32.46 (17.29) {26}	28.33 (15.33) {24}	23.55 (18.77) {22}	*d* = .49, *p* = .001	*d* = .94, *p* < .001^b^	*d* = .46, *p* = .011	*d* = .25, *p* = .007	*d* = .28, *p* = .484
WL	37.41 (12.75) {32}	36.96 (14.35) {27}			*d* = .03, *p* = .579				
CALIS_adol_									
ICBT	13.54 (6.56) {35}	10.59 (7.65) {32}	8.93 (7.63) {27}	9.72 (8.55) {25}	*d* = .41, *p* = .007	*d* = .50, *p* = .002^b^	*d* = .21, *p* = .254	*d* = .22, *p* = .183	*d* = -.10, *p* = .663
WL	13.97 (6.73) {35}	12.42 (8.65) {31}			*d* = .20, *p* = .180				
CALIS_mother_									
ICBT	32.14 (11.36) {35}	22.49 (12.67) {35}	23.21 (16.33) {33}	20.61 (14.96) {31}	*d* = .80, *p* < .001	*d* = .87, *p* < .001^b^	*d* = .93, *p* < .001	*d* = -.05, *p* = .856	*d* = .17, *p* = .450
WL	33.71 (11.91) {35}	35.34 (13.47) {32}			*d* = -.13, *p* = .513				
CALIS_father_									
ICBT	29.42 (14.07) {31}	25.89 (15.58) {27}	24.21 (14.53) {24}	16.59 (13.50) {22}	*d* = .24, *p* = .049	*d* = .93, *p* < .001^b^	*d* = .20, *p* = .227	*d* = .11, *p* = .195	*d* = .54, *p* = .044
WL	28.91 (9.74) {32}	28.52 (13.00) {27}			*d* = .03, *p* = .574				
WHO-5									
ICBT	44.69 (21.44) {35}	49.50 (21.69) {32}	55.85 (25.21) {27}	48.48 (25.98) {25}	*d* = .22, *p* = .143	*d* = .16, *p* = .129^b^	*d* = -.04, *p* = .945	*d* = .27, *p* = .100	*d* = -.29, *p* = .105
WL	48.91 (19.19) {35}	54.06 (20.39) {31}			*d* = .26, *p* = .135				
S-MFQ_adolescent_									
ICBT	10.46 (6.47) {35}	8.06 (7.77) {32}			*d* = .34, *p* = .014		*d* = -.11, *p* = .932		
WL	10.94 (7.07) {35}	7.77 (7.14) {31}			*d* = .45, *p* = .010				
S-MFQ_mother_									
ICBT	9.91 (5.79) {35}	5.77 (5.25) {35}			*d* = .75, *p* < .001		*d* = .60, *p* = .008		
WL	10.43 (6.15) {35}	9.47 (6.37) {32}			*d* = .15, *p* = .360				
S-MFQ_father_									
ICBT	8.26 (5.43) {31}	7.15 (5.86) {27}			*d* = .20, *p* = .103		*d* = .07, *p* = .813		
WL	7.88 (4.89) {32}	6.56 (4.85) {27}			*d* = .27, *p* = .229				
SEQ-C_total_									
ICBT	70.71 (13.88) {35}	75.25 (16.55) {32}			*d* = .30, *p* = .008		*d* = .12, *p* = .367		
WL	71.23 (15.76) {35}	74.00 (16.27) {31}			*d* = .17, *p* = .174				
SEQ-C_academic_									
ICBT	27.17 (7.14) {35}	27.13 (8.04) {32}			*d* = -.01, *p* = .970		*d* = -.08, *p* = .449		
WL	26.11 (8.49) {35}	26.71 (7.84) {31}			*d* = .07, *p* = .270				
SEQ-C_social_									
ICBT	25.09 (5.46) {35}	26.06 (6.11) {32}			*d* = .17, *p* = .133		*d* = .04, *p* = .668		
WL	26.20 (6.79) {35}	27.06 (6.32) {31}			*d* = .13, *p* = .412				
SEQ-C_emotional_									
ICBT	18.46 (5.92) {35}	22.06 (5.87) {32}			*d* = .61, *p* < .001		*d* = .38, *p* = .026		
WL	18.91 (5.26) {35}	20.23 (6.35) {31}			*d* = .23, *p* = .205				

*Note*. ADIS: Anxiety Disorder Interview Schedule for DSM-IV; CSR: Clinical Severity Rating; SCAS-C/P: Spence Children’s Anxiety Scale Child/Parent version; CALIS: Child Anxiety Life Inference Scale; WHO-5: WHO-5 Well-being Index Questionnaire; S-MFQ: short version of the Moods and Feelings Questionnaire; SEQ-C: The Self-Efficacy Questionnaire for Children; f/up: follow-up.

^a^ Pre to 3-month follow-up

^b^ Pre to 12-month follow-up. For the SCAS, CALIS and S-MFQ, a family-wise Bonferroni-corrected significance level of *p* = 0.017 was deployed. Effect size calculations based on observed values and expressed as Cohen’s *d*. Positive effect sizes indicate improvement.

**Fig 2:**
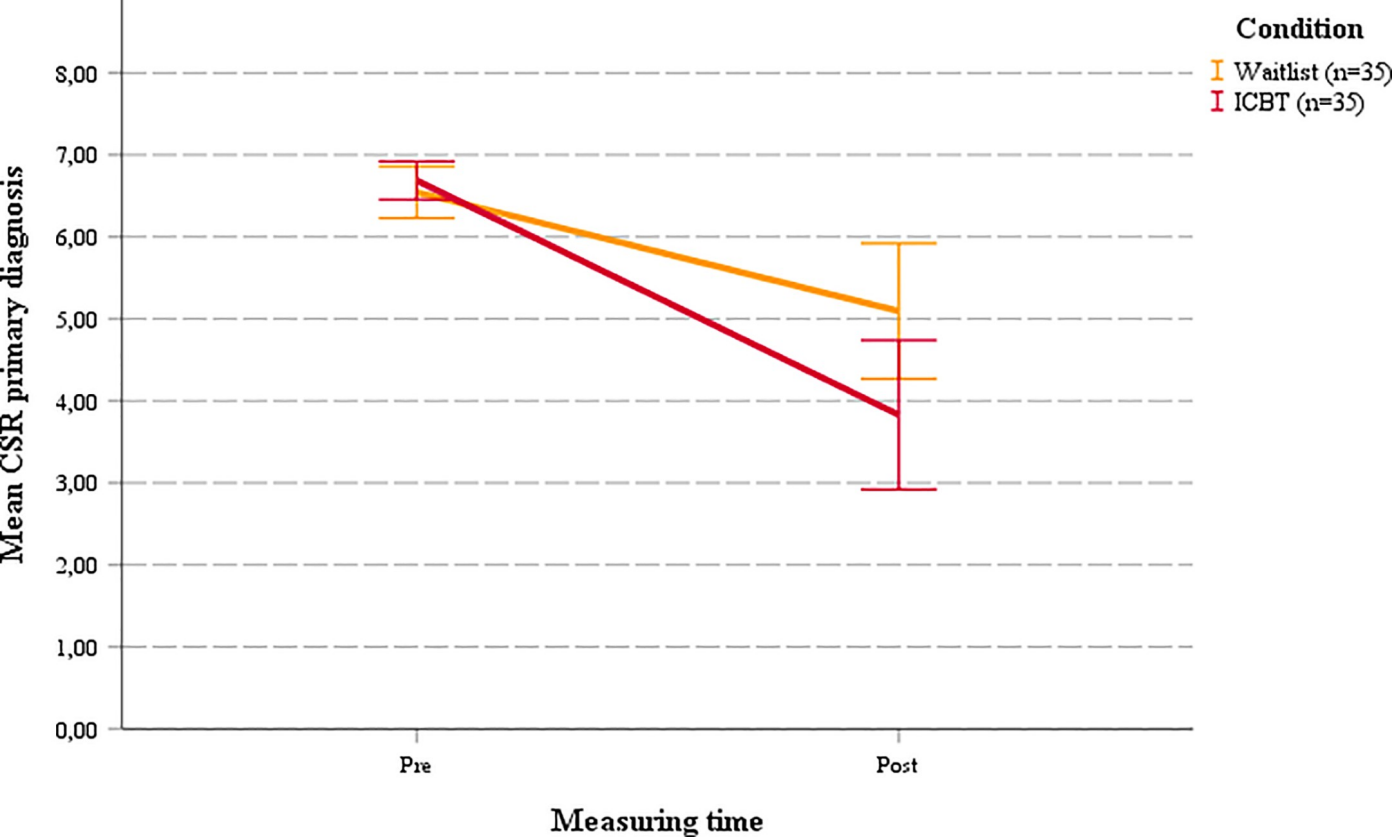
Mean CSR pre-post for primary diagnosis.

**Fig 3:**
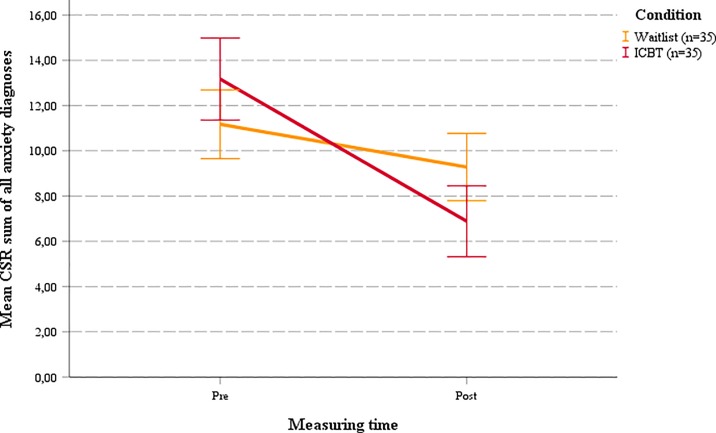
Mean CSR pre-post for sum of all anxiety diagnoses.

Pre-post MLM analyses revealed significant effects on clinician-rated severity of primary diagnosis (CSR_prim_; *d* = 0.65, *p* = 0.022) and all diagnoses (CSR_all_; *d* = 0.83, *p* = 0.002) (see Table 4 and Figs 2 and 3), demonstrating significantly larger improvements in diagnostic severity in the ICBT group compared to WL. For anxiety symptoms, pre-post comparisons on the SCAS revealed significant time × condition interactions for adolescents (*d* = 0.68, *p* < 0.001), mothers (*d* = 1.12, *p* < 0.001) and fathers (*d* = 0.46, *p* = 0.011), indicating significantly greater reductions in adolescent anxiety symptoms in the ICBT group compared to WL.

### Secondary outcomes

Significant time × condition interactions were found for mother reports on life interference (CALIS; *d* = 0.93, *p* < 0.001), mother reports on symptoms of depression (S-MFQ; *d* = 0.60, *p* = 0.008) and adolescent report on the emotional subscale of self-efficacy (SEQ-C; *d* = 0.38, *p* = 0.026). For the remaining secondary outcome measures (including adolescent and father reported CALIS, adolescent and father reported S-MFQ, WHO-5, SEQ-C total scale, and SEQ-C academic and social subscales), no significant interaction effects were found (see Table 4).

### Improvement and recovery rates

According to adolescent reported anxiety symptoms on SCAS-C, significantly more participants in the ICBT condition (69%) than in the WL condition (26%) were classified as improved at post-treatment (*χ*^2^ (1) = 11.62, *p* = .001). Also, significantly more participants in the ICBT condition (44%) than in the WL condition (6%) were classified as recovered (*χ*^2^ (1) = 11.56, *p* = .001) according to adolescent SCAS-C report. For the post-treatment mother-reported SCAS-P, significantly more participants in the ICBT condition (69%) than in the WL condition (22%) were considered improved (*χ*^2^ (1) = 14.66, *p* < .001). There were, however, no significant differences in number of participants classified as recovered (*χ*^2^ (1) = 4.62, *p* = .032) in the ICBT condition (26%) compared to the WL condition (6%). For father reported SCAS-P, the number of participants improved post-treatment in the ICBT condition (35%) was not significantly larger than those improved in the WL condition (19%, *χ*^2^ (1) = 2.02, *p* = .156). Similarly, participants classified as recovered according to father reported SCAS-P at post-treatment did not differ significantly (Fisher’s exact test: *p* = 1.00 (two sided)) in the ICBT group (4%) compared to the WL group (7%).

### Treatment maintenance

At 3-month follow-up, 36% adolescents were free of their primary anxiety diagnosis and 30% were free of all anxiety diagnoses. MLM within-group analyses of the CSR_prim_ and CSR_all_ from pre-treatment to 3-month follow-up showed large and significant improvements (*d* = 1.50, *p* < 0.001; and *d* = 1.36, *p* < 0.001, respectively), while analyses from post to 3-month follow-up revealed small and non-significant changes (*d* = -0.02, *p* = 0.495; and *d* = 0.11, *p* = 0.482, respectively).

**Fig 4:**
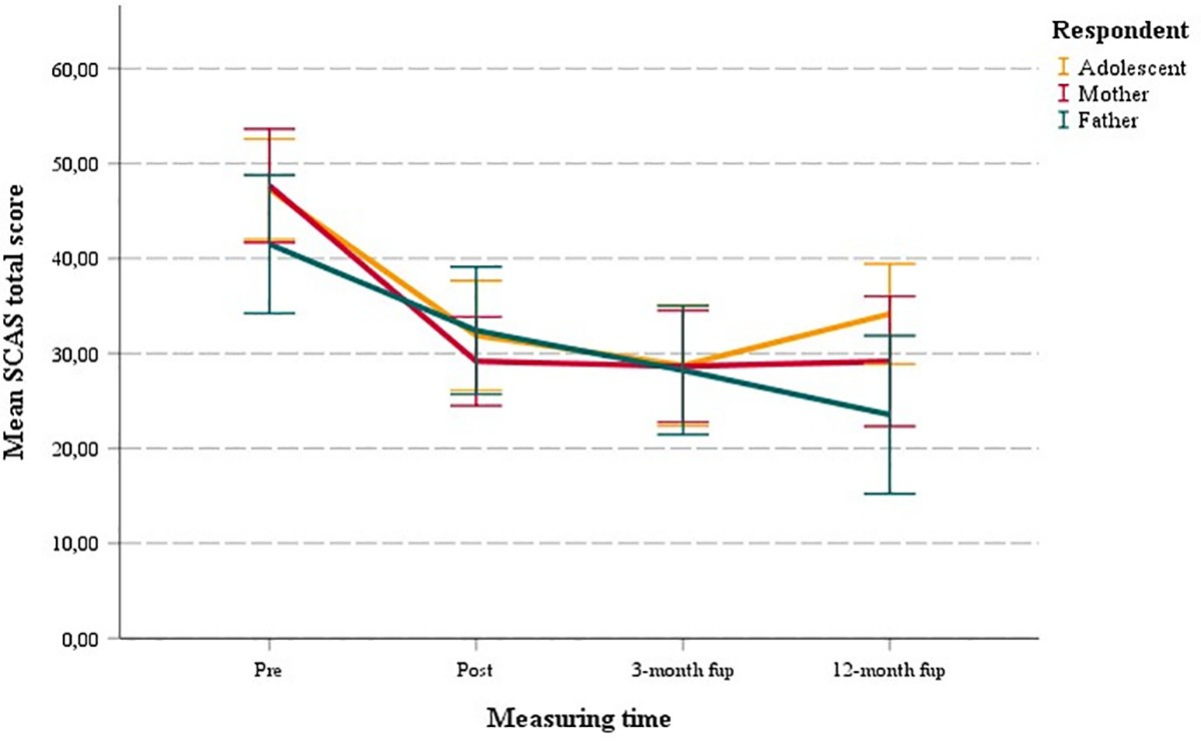
Mean SCAS total scores at pre, post, 3- and 12-month follow-up for adolescents, mothers and fathers.

Within-group analyses of the SCAS reports from pre to 12-month follow-up (see Fig 4 and Table 4) revealed large and highly significant improvements for all responders (adolescents: *d* = .99, *p* < .001; mothers: *d* = 1.10, *p* < .001; and fathers: *d* = .94, *p* < .001). Analyses from post to 3-month follow-up revealed significant improvements for the father report on SCAS-P (*d* = 0.25, *p* = 0.007). Otherwise, analyses generally showed non-significant positive changes in the follow-up periods.

At 3-month follow-up, the number of adolescents classified as improved on the SCAS was 67% according to adolescent report, 70% according to mother report and 61% according to father report. The number classified as recovered was 52% according to adolescent report, 39% according to mother report, and 30% according to father report. At 12 month follow-up, the number of adolescents categorized as improved on the SCAS was 48% according to adolescent report, 65% according to mother report, and 67% according to father report. The number classified as recovered was 40% according to adolescent report, 45% according to mother report, and 43% according to father report.

For the CALIS reports, progress was also maintained during the follow-up period according to adolescent, mother and father reports at 3- and 12-month follow-up, with within-group analyses from pre to 12-month follow-up showing significant improvements of moderate to large ES’s (adolescents: *d* = 0.50, *p* = 0.002; mothers: *d* = 0.868, *p* < 0.001; and fathers: *d* = 0.931, *p* < 0.001). Analyses of participants’ emotional well-being scores on the WHO-5 indicated no change, as the total treatment effect from baseline to 12-month follow-up was small and non-significant (*d* = 0.16, *p* = 0.129). Similarly, effects for both follow-up periods analyzed separately were small and non-significant (see Table 4).

### Treatment satisfaction

Overall, adolescents were satisfied with the intervention, with a mean of 17.3 (SD = 3.64) out of a maximum score of 21. Seventeen (55%) rated the statement “the treatment helped me” to be ‘true’, nine (29%) to be ‘partly true’, and five (16%) not to be true. Eighteen adolescents (58%) would recommend the treatment to a friend, four (13%) would not recommend it, and the remaining nine (29%) answered ‘partly true’ to the statement. Adolescents’ qualitative comments on the treatment were generally highly positive. Two (7%) would, however, have liked more time, and one (3%) expressed the need for face-to-face sessions with the therapist. Mothers and fathers were also generally satisfied with the intervention with a mean rating of 23.1 (SD = 3.66) and 22.2 (SD = 3.19), respectively, out of max 27.

Participants and their parents were also asked post-treatment whether the treatment had caused them/their child to feel worse. One adolescent (3%) rated the statement to be true, while three adolescents (10%) rated it to be ‘partly true’. One mother (3%) rated the statement to be true, while two mothers (6%) answered ‘partly true’ to the statement. No fathers rated the statement to be ‘true’, but three (12%) rated it to be ‘partly true’. Regarding those (n = 2) who rated the above statement to be true, additional qualitative information from questionnaires and booster-sessions was collected but did not indicate requirements of further clinical intervention.

## Discussion

The present study evaluated the efficacy of therapist-guided ICBT for adolescents with anxiety disorders. To the best of our knowledge, the study is the first randomized evaluation of the ChilledOut Online program, and it is the first systematic evaluation of an ICBT intervention for this population in Denmark.

Lending support to our main hypothesis, participants receiving ICBT demonstrated significant improvements at post-treatment compared to participants in the WL condition across all raters on diagnostic severity and level of anxiety symptoms (CSR and SCAS). The between-group ES’s found for CSR_prim_ and CSR_all_ in the present study (*d* = 0.65 and *d* = 0.83, respectively) are in the lower end of those found in other similar WL controlled studies; for instance by Lenhard et al. [48]: *d* = 0.69; and Vigerland et al. [83]: *d* = 1.66. In their study of the Cool Teens CD-ROM, Wuthrich et al. [56] showed a between-group ES, *d* = 1.35. The within-treatment pre-post effects on diagnostic severity were, however, quite similar in our study to that of other studies; for instance a *d* = 1.48 on CSR_prim_ compared to *d*s 1.32–1.69 [56, 83, 84]. The lower between-group ES in the present study may be explained by the large improvement in the WL group, *d* = 0.83. Seven of the 35 WL participants (20%) reported having had contact with mental health services during the WL period, three had received hypnosis in private practice, one CBT with the GP, and three had contacted the local psychiatric hospital, possibly explaining part of the improvements found in the WL group. Large changes in waitlist participants is however not unprecedented as previous studies of CBT with youth/children has found similar outcomes (e.g., [85]). Despite these improvements, most WL participants (32 of 35) received ICBT after the waitlist period.

The study demonstrated significant treatment effects on both the adolescent and parent ratings of anxiety symptoms on SCAS, but with large differences in the size of the effect: SCAS-C *d* = 0.68; SCAS-P_mother_ *d* = 1.12; and SCAS-P_father_ *d* = 0.46. Moderate between-group treatment effects for child and adolescent self-rated anxiety symptoms on SCAS-C has previously been shown [84] but from an overall perspective on the literature, these self-ratings tend to fluctuate from small and/or non-significant [46, 48, 83] to large and highly significant [44]. It may be that the SCAS-C does not adequately frame the symptoms as experienced by adolescents. It may also be a testimony of the adolescents’ emotional self-awareness being under development with accompanying variances in their ability to detect and report changes. The outcome on mother reported SCAS-P (*d* = 1.12) is, on the other hand, larger than those reported in previous studies (*d* = 0.16–0.69; [46, 48, 56, 83, 84, 86]). It is not common for CBT studies to measure both mother- and father-reported anxiety. The present study is, to the best of our knowledge, the first RCT of ICBT to include separate reports from both parents. However, a similar difference in parents’ ratings of anxiety symptoms was found in a prior study of face-to-face CBT with children and adolescents at CEBU [87] with mothers reporting larger improvements than fathers. More research is needed to further investigate the divergence of anxiety symptom scores between adolescents, mothers and fathers.

Results of the study partially supported our second hypothesis, that the ICBT treatment would reveal better outcome in depressive symptoms, self-efficacy and quality of life compared to the WL condition. Significant improvements were found on the mother ratings of depressive symptoms (S-MFQ), mother ratings of anxiety life interference (CALIS) and self-ratings of the emotional self-efficacy sub-scale (SEQ-C emotional). Two previous ICBT studies treating adolescents with anxiety disorders also measured depressive symptoms, with one [48] showing no effects and the other [44] demonstrating large effects (*d* = 1.39). As was found in this study, it is not unusual to find smaller outcomes (and/or less frequently significant findings) on depression than on anxiety symptoms (e.g., [88]) in studies using CBT to treat anxiety disorders.

Supporting our third hypothesis, results at 3- and 12-month follow-up assessment demonstrated a maintenance of treatment effects for all included measures. These results are similar to those of Wuthrich et al. [56], Spence et al. [84], and Tillfors et al. [44], who showed treatment maintenance at 3-, 6-, and 12-month follow-up, respectively. However, our results differ from those of Lenhard et al. [48], Vigerland et al. [83], and Spence et al. [46], whom have all demonstrated further anxiety symptom improvements during the follow-up periods of 3-, 3- and 12-month, respectively. Future studies including longer follow-up periods might help illuminate how improvements achieved through ICBT for children and adolescents evolve over time.

### Limitations

The study has a number of limitations that should be considered when interpreting the results. Although improvements in the ICBT group suggests that recovery could be attributed to the specific intervention and not to confounding factors such as spontaneous remission or repeated assessment, an active control condition would be required to rule out potential effects of such unspecific factors. The study was not controlled in the follow-up period since participants in the WL condition received treatment during this period. Rater blindness to treatment condition was often broken post-treatment and at follow-up. Moreover, the use of self-referral and the high socioeconomic status of participant families could have implications for generalizability of results, although it is difficult to predict the direction of potential effects. Lastly, the majority of CSR ratings were based on ADIS interviews conducted by students. Although thoroughly trained and supervised, they had only modest prior assessment experience compared to trained psychologists used in other studies. Combined with low variability in CSR of primary diagnosis (all were scored 6–8) and the use of telephone-recorded interviews in the reliability checks, this might help explain the moderate inter-rater reliability scores found in the study.

## Conclusion

This study demonstrated the Danish version of ChilledOut Online to be efficacious and feasible in relieving symptoms of anxiety in adolescents. As such, the study supports previous findings of similar guided ICBT interventions and helps build a strong foundation for future research in and implementation of ICBT in mental health services for adolescents with anxiety disorders.

## Supporting information

S1 File CONSORT checklist. (PDF)

S2 File Trial study protocol (ethics committee). (PDF)

S3 File Trial study protocol_Danish version. (PDF)
