# Supplementary material for: A randomized controlled trial examining the efficacy of an internet-based cognitive behavioral therapy program for adolescents with anxiety disorders
Source: PLoS One. 2019 Sep 18;14(9):e0222485. doi: 10.1371/journal.pone.0222485 (PMC6750608; doi:10.1371/journal.pone.0222485)
Supplement: S2 File — (PDF) [file pone.0222485.s002.pdf]

A randomized controlled trial of internet-based treatment for adolescents with anxiety disorders

Background

Anxiety is one of the most common mental disorders among children and adolescents (1,2); research shows that 2.5–5% of youths from Western countries fulfill the diagnostic criteria for an anxiety diagnosis while the lifetime prevalence is estimated to be between 15 and 20% (1,3-5). The presence of an anxiety disorder has significant negative consequences for the individual child including problems at school, with peers and in the family (6,7) and many anxiety disorders are thought to have a chronic developmental course causing the anxiety to last through adolescence to adulthood if it is not treated (2,8-10). Furthermore, the presence of anxiety early in life can be a marker for the later development of other mental disorders such as depression and substance abuse (4,11,12). Thus, anxiety disorders becomes an expensive societal problem (13). Research suggests however, that only a small proportion of children and adolescents with anxiety disorders receive professional treatment (14-17). Apart from a very limited range of available treatments for children and adolescents, the lack of treatment may also be caused by help-seeking reluctance due to fear of social stigma, confidentiality and privacy concerns and possible preference for self-reliance (18).

Cognitive behavioral therapy (CBT) is a well-documented and effective treatment method for children and adolescents with anxiety disorders (19-24). A recent meta-analysis (22) of 55 randomized controlled trials (RCTs) of CBT with youths aged six to nineteen with anxiety disorders (n = 4256) reported a mean effect size of d = .77 compared to waitlist control groups and a mean effect size of d = .39 when comparing CBT to active controls (e.g. attention placebo, relaxation training, or placebo medication).

Internationally, there has been an increase in the development and implementation of internet-based CBT (ICBT) programs over the past few years as means to reduce costs and increase access to the psychological treatment of anxiety disorders (25,26). Using the internet and computer/tablet/smartphone may hold potential to overcome some of the aforementioned help-seeking barriers by offering greater anonymity, easy accessibility in terms of both time and place of the treatment, greater flexibility in terms of treatment pace and specific focus, as well as larger appeal to children and adolescents through the use of interactive and stimulating program elements (27-29).

ICBT has proven effective in treating adults with anxiety disorders; the effects found corresponds to those demonstrated in therapist administered CBT (30-34). There are, however, relatively few RCTs of ICBT for anxious children and adolescents. To the best of our knowledge, currently three RCTs of ICBT for anxious children (aged 7-14) (35-37), three RCTs of ICBT for anxious adolescents (aged 12-21) (38-40), and two RCTs of ICBT for anxious and depressed adolescents (aged 11-21) (41,42) can be localized. In all studies ICBT has proven superior to waitlist controls and nearly as effective as therapist administered CBT. The studies originate from USA (35), Australia (36-39,42), Great Britain (41), and Sweden (40). No ICBT program for children or adolescents with anxiety disorders has yet been developed nor evaluated in Denmark.

ICBT may be particularly appropriate for adolescents (1) as the therapeutic content can be accessed at any time and thus can be flexibly adapted to suit the adolescents' daily commitments and activities, (2) because

ICBT offers more privacy, greater anonymity and confidentiality, and less social stigma compared to regular face-to-face therapy, characteristics that are greatly appreciated by adolescents (43,44), and (3) because most adolescents are comfortable with and highly skilled users of computer technologies (45). According to a recent report from EU Kids Online (46) based on more than 25,000 youths (aged 9-16) and their parents from 25 European countries including Denmark, the use of the internet can now be considered an integrated part of children's everyday lives. Ninety-three percent of the youths included in this study accessed the internet at least once a week; for 80% of those aged 15-16 it happened on a daily basis. On average, participants spent 88 minutes every day online. Thus, ICBT for Danish adolescents may hold great potential.

Aim

The aim of this project is through a waitlist controlled RCT to evaluate the feasibility and efficacy of a Danish version of the ICBT program ChilledOut Online, recently developed at Macquarie University, Sydney, Australia, for adolescents aged 13 to 17 with anxiety disorders.

The study is an extension of a former research project, *Evidence based treatment of youths with anxiety disorders*, conducted at the Centre for the Psychological Treatment of Children and Adolescents (CEBU), Department of Psychology and Behavioral Sciences, Aarhus University (project id: M-20110019), in which we investigated the efficacy of the Danish version of the manualized group-treatment program Cool Kids with good results (48.2% of participants were free of all anxiety diagnoses at post-treatment compared with 5.7% in the waitlist condition) (47).

Project hypotheses

We expect the ChilledOut Online intervention to be well received by the adolescents as it is thought to fit well into their daily routines and general life style.

Based on previous reviews and meta-analyses of comparable ICBT programs for children and adolescents (48-5), ChilledOut Online is expected to have a significant positive effect on participants' anxiety symptoms (as measured by ADIS, SCAS, and CALIS, elaborated below) as well as on their general mental health (as measured by S-MFQ, WHO-5, and SDQ, elaborated below) compared to the waitlist control group.

Through benchmarking analyses ChilledOut Online is expected to be nearly as effective as comparable therapist administered group-treatment programs (53,54).

We expect a maintenance of the treatment effect at 3-month follow-up.

Participants' age and specific anxiety diagnoses (as measured by the ADIS), the quality of the therapeutic alliance (as measured by the WAI-S), degree of self-efficacy (as measured by SEQ-C), as well as degree of parent involvement (measured as their self-reported average amount of weekly time spent on program relevant activities) are expected to partly moderate or mediate treatment effect.

Methods

Study design

The study is designed as an RCT with 70 adolescents randomly allocated to two groups:

1) ICBT (n = 35)

2) Waitlist control (n = 35)

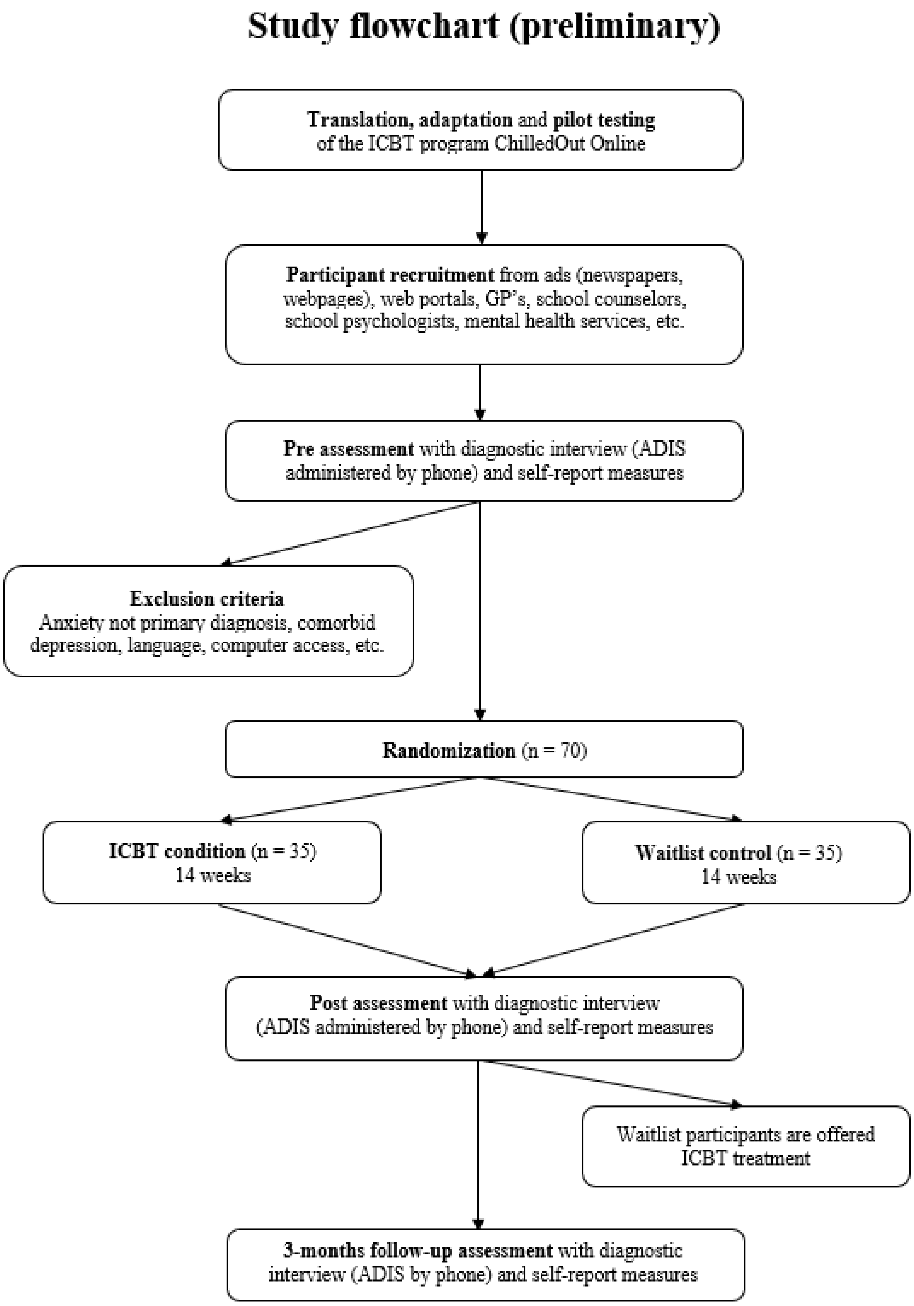

Participants

Participants will be recruited primarily from the CEBU waiting list as well as from advertisements in the CEBU newsletter, on the CEBU website, at Aarhus University's website, at *Angstforeningen*'s website, in newspapers, on selected Facebook pages (the personal pages of Silke Stjerneklar and Mikael Thastum, on *Børn med angst – forum for forældre*, on *Angstforeningen*, and on *Danske psykologer*), through study counsellors and school psychologists around the country as well as referrals from professionals. The recruitment is planned to take place from June through October 2015.

Generally, the research literature demonstrates large effect sizes when comparing active treatments and control groups with no intervention (36). Power calculations based on t-test of the difference between two independent means and previous studies of comparable ICBT programs for children and adolescents with anxiety disorders has suggested that a total sample size of 52 will provide power of .80 to detect a large effect size (d = .80) at α = .05. Thus, a sample of 70 is assumed able to withstand attrition of 2-13% (38-40) in addition to detect a clinically meaningful difference in effect between the two conditions (ICBT versus wait-list).

In- and exclusion criteria

Seventy adolescents aged 13 to 17 with a primary anxiety diagnosis (Separation anxiety disorder, generalized anxiety disorder, social anxiety disorder, specific phobia, obsessive compulsive disorder, and panic disorder) according to DSM-IV (55) will be included in the study. Participants must have direct access to a home computer with internet and be able to read and write in Danish. Exclusion criteria are: PTSD as primary diagnosis, severe comorbid depression (Clinical Severity Rating (CSR) > 6) as measured with the Anxiety Disorders Interview Schedule for DSM-IV, Child and Parent Versions (ADIS-IV C/P) (56), substance abuse, current severe self-harm or suicidal ideation, pervasive developmental disorder or learning disorder, intellectual disability, and psychotic symptoms. Participants are encouraged not to make changes to their medication status during the trial.

Procedure

Participants on the CEBU waiting list are contacted via telephone by a student therapist from CEBU. During this conversation the time and date for diagnostic interview is set. Subsequently, a letter is sent to the family with a confirmation of the agreed time and date, the interview procedure is elaborated, and the families are informed of the procedures to follow.

Shortly thereafter, the adolescent, his/her mother and father receives a link to electronic self-report questionnaires, which they are asked to complete as soon as possible. Families failing to complete the questionnaires receives weekly e-mail reminders. After three weeks the families are contacted via telephone by the appointed therapist.

Diagnostic status will be assessed separately with the adolescent and one of the parents with the ADIS-IV C/P (56) administered over the telephone, which has yielded high reliability and validity comparable to that of face-to-face administration (57,58). The interviews are conducted by graduate students working at CEBU, trained in using the ADIS-IV and under regular supervision by clinical psychologists. All interviews are recorded with the purpose of re-assessing diagnostic reliability at post-hoc ratings (20%).

After the initial assessment included participants are randomized to ICBT or waitlist condition with a fixed block size of 10 (5/5, respectively). The randomization sequence will be created with an online computer algorithm, stored and administered by an external secretary at the University. Participants randomized to the control condition will be offered the ICBT intervention after their 14 weeks waiting period. All participants will have access to the program for at least three months after treatment.

The families will be notified of their in- or exclusion from the study as well as of their group allocation (ICBT or waitlist) within one week after their diagnostic interview. Subsequently, adolescents allocated to the ICBT condition will receive a letter with the program URL, a personal username, a temporary password, the treatment start date as well as a calendar plan of the future therapist phone calls. Also, this letter will include a short presentation of the appointed therapist and his or her e-mail address.

Participants' usernames are constituted by their first and last name. The temporary password is generated by an online password generator (Norton Identity Safe Password-generator). The program administrator will enter participants' user profiles (including name, date of birth, e-mail address, gender, access expiration date, username and password) manually into the program back end. After initial login participants will automatically be asked to change their password. This is done to ensure that only the adolescents themselves are familiar with their credentials. Besides the adolescents, only the appointed therapist and the program administrator will be able to access participants' program interface (i.e. have access to completed worksheets, program activity overview, etc.) through a function (*Impersonating*) in the program back end. The therapists will inform the adolescents of these details during their first conversation.

Treatment

The internet-based treatment program ChilledOut Online was originally developed at the Centre for Emotional Health, Macquarie University, Australia, as an expansion of the manualized group-treatment program 'Chilled' for adolescents aged 13 to 17 (54,59) created by Professor Ron Rapee and colleagues. Mikael Thastum, head of CEBU, has gotten permission to translate, adapt and use ChilledOut Online as part of his ongoing research collaboration with Professor Rapee. The program is designed for use on the adolescents' home computer under continuous support and guidance from their parents. The program is based on a cognitive behavioral skills training approach and includes components such as cognitive restructuring, graded exposure, response prevention, behavioral experiments, problem solving, awareness exercises and assertive behavior.

ChilledOut Online consists of eight online modules of approximately 30 minutes each to be completed over a 12 to 14-weeks period. Using interactive examples, exercises and information videos delivered via a combination of multimedia formats (i.e. text, audio, illustrations, cartoons, and video vignettes) the adolescents are taught a range of CBT-inspired strategies to help them manage and reduce their anxiety.

The headlines of the eight program modules are:

1. Understanding anxiety: Introduction to the program functions and psychoeducation about anxiety
2. Setting goals: Guides the adolescents in setting realistic goals for the treatment course, introduces rewards, self-rewards and the balancing of expectations related to the intervention
3. Realistic thinking I: Focuses on linking thoughts and feelings, and introduces cognitive restructuring as realistic thinking
4. Stepladders I: About facing your fears and managing fears by developing, planning and executing stepladders

5. Stepladders II: On how to revise stepladders and doing behavioral experiments
6. Realistic thinking II: Revises inner realistic thinking, acting 'as if' and awareness training (called 'worry surfing')
7. Other coping skills: Focuses on skills such as problem solving, constructive feedback, assertive communication/behavior, and relaxation
8. Staying chilled: General summary of the program and relapse prevention

Embedded in the program interface are a variety of tools and features that the adolescents can access through menu items at the top of the screen. These features include profile information, program use (e.g. number of logins), a graph depicting the adolescent's progress based on a short weekly questionnaire, a diary, worksheets, a message function enabling participants to receive help from the program administrator on technical issues, a link to a help page with contact information in case of emergency, and a page containing a broad description of the program terms and conditions.

Parents receive a booklet, the ChilledOut Parent Companion, introducing the core components of CBT and advice on how to best support their adolescent in the application of the skills learned through the program. Parents are encouraged to talk regularly with their adolescent about the program and to support the adolescent in his or her efforts to complete their weekly exercises. Furthermore, parents are invited to sign up for a closed online network in which they are able to ask questions, give good advice, exchange experiences, etc. with other parents who's adolescents are also included in the trial. Activities in the online network will be closely supervised by the project manager to ensure that contributions and postings are relevant and in a good tone. In the instance of a post not meeting these requirements the responsible parent will be contacted by the project manager and instructed in the network purpose and regulations. With the online network invitation parents also receive a consent form in which they give permission for CEBU to use anonymized parts of the network postings in future research activities. Returning the signed consent form will be a prerequisite for inclusion in the online network. Parents' participation in the network is voluntary and will not affect their participation in the study.

Once a week the adolescents will receive a short phone call from their therapist. The purpose of this call is to ensure participants' understanding of the treatment rationale, to assist the learning and application of program skills and strategies in the adolescents' daily lives, as well as to support problem solving of any difficulties that may arise throughout treatment. The therapists will be graduate psychology students from CEBU, trained in CBT and supervised by clinical psychologists. Moreover, the therapists will be equipped with a detailed manual, based on a recent pilot trial of the intervention. All phone calls will be recorded to ensure therapist integrity and for supervision purposes. Participants will be encouraged to contact their therapist via e-mail in case they have difficulties or questions regarding their program completion. All inquiries from participants will be answered within two working days. The non-acute properties of these e-mail correspondances will be highlighted with all participants. In case of emergency participants are referred to the hotline phone numbers on the web site.

At three-month follow-up, adolescents will receive a booster phone call from their therapist. The aim of this conversation is to support the adolescents' continued work with their learned skills as well as to help them solve any difficulties that the families may have encountered since treatment completion. Based on the adolescent's progress and overall well-being, the therapist will make an evaluation during the booster

call of whether the adolescent should be granted a three month extended web site access or not. Unfortunately, unlimited access to the web site is not possible within the framework of this study, as access should always be supported by the program administrator. The availability of the program administrator is limited by time- and financial constraints.

Measures and data

The study will use a variety of self-report questionnaires comparable to those previously administered at CEBU (listed in appendix 1). Questionnaires are delivered via e-mail at pre, after week 4 and 8, post treatment, at 3- and at 12-month follow-up, and they are completed separately by adolescents, mothers and fathers. The questionnaires are administered through an electronic data collection platform, SurveyXact (http://www.surveyxact.dk) and imported to IBM® SPSS® statistics (Armonk, NY: IBM Corp.), where they will be stored on a secure drive. Clinical ratings will be performed by clinical psychologists and research assistants.

Data storage

All information concerning participating families are being protected according to the current national regulations for the processing of personal data and legal status of patients. Data (including patient files, program usernames and passwords) will be stored at CEBU, Department of Psychology and Behavioral Sciences, Aarhus University, under the legal responsibility of PhD-fellow Silke Stjerneklar and Professor Mikael Thastum.

Statistical analyses

Most statistical analyses will be 'intention to treat'. Thus, drop-outs will receive the intended questionnaires at all measuring points and will be invited to take part in the diagnostic telephone interviews.
Analyses of drop-outs: sociodemographic data, specific diagnoses etc. will be gathered and compared to study completers to ensure the generalizability of results.
Baseline group comparison: baseline data from the two groups (ICBT and waitlist) will be compared using t-test and chi^2 to evaluate the result of the randomization.
Primary outcomes: the main effect of the intervention will be statistically evaluated using mixed-model repeated-measures ANOVAs (time x condition). Statistically significant results will be further analyzed with post-hoc-tests/planned contrast analyses.
Results will be benchmarked with results from comparable studies (including the previous RCT study from CEBU).

Ethical considerations

No ethical issues are expected from the present trial. Ethical approval will be attained from the local Ethics Committee of Central Denmark Region and from the Danish Data Protection Agency.

The project group is fully aware of the ethical issues possibly related to the inclusion of minors in scientific health studies. The inclusion of participants aged 13 to 17 in the present study is considered necessary for the project completion as the treatment program under investigation was developed for this specific population. We expect the ChilledOut Online treatment to contribute to a significant reduction in participants' mental health issues, especially in those related to anxiety symptoms. Furthermore, we expect the intervention to yield minimal negative side effects. The program is expected to fit well with the adolescents everyday lives and to be well received by them. In accordance with current legislation, specific participant information material has been developed for participants aged 15 to 17.

Study participation is voluntary and participating families can discontinue their participation at any time without it affecting any future treatments they might be interested in seeking outside CEBU. Families deciding not to be included in the study will not be offered other treatments, as CEBU has no legal treatment obligation.

Participating families are informed of the study procedures, purpose and methods both verbally and in writing, and they are required to sign a consent form before treatment start. Participants will receive no economic compensation for their participation in the study.

CBT is a broadly recognized and well established therapeutic method, also when offered online, and there are no known negative side effects, risks or disadvantages. The use of a waitlist control group is done to obtain vital data for research control purposes. Those randomized to the waitlist control condition will be offered the ICBT treatment after the end of the 14-weeks waiting period.

The current Danish law on the processing of personal data will be obeyed.

Regardless of the outcome, study results will be attempted published in an international journal. Single participants will not be identifiable in published data.

Obtaining informed consent

Contact with potential project participants will be initiated either through the CEBU web site or through advertisements in various media. Interested families are invited to send in a brief description of their adolescent’s difficulties via e-mail. Families deemed eligible for inclusion in the study are offered a place on the Centre waiting list and sent a leaflet with information on what to expect if they are included in the study (e.g. the randomization procedure and the ‘no treatment’ waiting period if allocated to waitlist). When families consent to the circumstances described in the leaflet, they are signed up for a possible spot in the trial.

From August 1st, 2015 to December 31st, 2015 (within which the diagnostic interviews will take place) families on the waiting list will be contacted via telephone by a student therapist from CEBU, agreeing on a time and date for the diagnostic interview. During this conversation, the adolescents and parents are verbally informed of the project. The students making these phone calls are psychology graduate students in the last year of their education, under continuous supervision and experienced in communicating with children and adolescents. The information will be delivered in a respectful manner and adapted to the parents’ and the adolescents’ individual prerequisites for understanding and considering the information given.

The project group is aware that verbal information regarding participation in scientific studies should generally be delivered face-to-face. This despite, we choose to deliver the verbal information via telephone as it aligns with the entire study rationale, i.e. internet-based treatment. One of the main arguments for testing this type of treatment is, that participants are exempted from several of the help-seeking barriers surrounding regular face-to-face therapy, e.g. travel time and high economic costs. Our hope is, that the present intervention can represent a national, easy assessable, cost saving treatment option for patients and for the surrounding health care system. Something that we will not be able to investigate to the same degree, if participants are required to be physically present for an introductory meeting at CEBU. We worry that such a meeting may exclude less resourceful families; the same families that are also excluded from the current treatment options offered through the national health care system. In the instance that individual families

express the need for an introductory face-to-face meeting we are more than happy to make the arrangements.

After having received the verbal project information an invitation letter is send to the family confirming the date and time for the diagnostic interview, and explaining the study procedures to follow. Included in this letter is a deputy consent form (in case of shared custody informed consent is required from both parents separately), consent forms regarding audio recording, information exchange, and publication of anonymous data, written participant information, a formal note on research subjects' rights, as well as a stamped envelope. Participants are asked to return the signed consent forms within three working days. The Centre secretary will call those that do not return the consent forms.

Project management and financial support

The project takes place at the Centre for Psychological Treatment of Children and Adolescents (CEBU), a research and teaching facility at the Department of Psychology and Behavioral Sciences, Aarhus University, Denmark. The project is developed in a collaboration between Silke Stjerneklar, MSc, PhD-fellow, and project manager; Mikael Thastum, Professor, PhD., and head of CEBU; and Esben Hougaard, Professor.

The project manager's salary is funded through a PhD-fellow scholarship from the Aarhus BSS Graduate School (BSS) at Aarhus University. The scholarship is effectuated from February 1st, 2014 to January 31st, 2017, not including possible leaves and extensions. The remaining costs for the project, including salaries for a clinical psychologist and the Centre secretary will be covered by a grant from Trygfonden.

References

1. Rapee, R.M., Schniering, C.A. & Hudson, J.L. (2009). Anxiety disorders during childhood and adolescence: Origins and treatment. *Annual Review of Clinical Psychology, 5*, 311-341.
2. Spence, S.H. (1998). A measure of anxiety symptoms among children. *Behavior Research and Therapy, 36*(5), 545-566.
3. Costello EJ, Mustillo S, Erkanli A, Keeler G, Angold A. (2003). Prevalence and development of psychiatric disorders in childhood and adolescence. *Arch Gen Psychiatry, 60*, 837-844.
4. Beesdo K, Knappe S, Pine DS. (2009). Anxiety and anxiety disorders in children and adolescents: Developmental issues and implications for DSM-V. *Psychiatr Clin North Am, 32*, 483-524.
5. Costello EJ, Egger HL, Copeland W, Erkanli A, Angold A. (2011). The developmental epidemiology of anxiety disorders: Phenomenology, prevalence, and comorbidity. In: *Anxiety disorders in children and adolescents. 2 ed.* Silverman WK, Field AP, Eds. Cambridge, Cambridge University Press, 56-75.
6. Davis TE, Ollendick TH, Nebel-Schwalm M. (2008). Intellectual ability and achievement in anxiety-disordered children: A clarification and extension of the literature. *Journal of Psychopathology and Behavioral Assessment, 30*, 43-51.
7. Angold A, Messer SC, Stangl D, Farmer EM, Costello EJ, Burns BJ. Perceived parental burden and service use for child and adolescent psychiatric disorders. Am J Public Health. 1998;88(1):75–80.
8. Kessler RC, Berglund P, Demler O, Jin R, Merikangas KR, Walters EE. (2005). Lifetime prevalence and age-of-onset distributions of DSM-IV disorders in the National Comorbidity Survey Replication. *Arch Gen Psychiatry, 62*, 593-602.
9. Muris, P. (2006). The pathogenesis of childhood anxiety disorders: Considerations from a developmental psychopathology perspective. *International Journal of Behavioral Development, 30*(1), 5-11.
10. Woodward LJ, Fergusson DM. Life course outcomes of young people with anxiety disorders in adolescence. J Am Acad Child Adolesc Psychiatry. 2001;40(9):1086–93.
11. Bittner A, Egger HL, Erkanli A, Jane CE, Foley DL, Angold A. What do childhood anxiety disorders predict? J Child Psychol Psychiatry 2007 Dec;48(12):1174-83.
12. Kovacs M, Gatsonis C, Paulauskas SL, Richards C. Depressive disorders in childhood. IV. A longitudinal study of comorbidity with and risk for anxiety disorders. Arch Gen Psychiatry 1989 Sep;46(9):776-82.
13. Bodden DH, Dirksen CD, Bogels SM. (2008). Societal burden of clinically anxious youth referred for treatment: A cost-of-illness study. *J Abnorm Child Psychol, 36*, 487-497.
14. Essau CA, Conradt J, Petermann F. (2000). Frequency, comorbidity, and psychosocial impairment of anxiety disorders in German adolescents. *J Anxiety Disord, 14*, 263-279.
15. Essau CA. Frequency and patterns of mental health services utilization among adolescents with anxiety and depressive disorders. Depress Anxiety. 2005;22(3):130–7.
16. Sawyer MG, Arney FM, Baghurst PA, et al. The mental health of young people in Australia: key findings from the child and adolescent component of the national survey of mental health and well-being. Aust N Z J Psychiatry. 2001;35(6):806–14.
17. Kataoka SH. Unmet need for mental health care among U.S. children: variation by ethnicity and insurance status. Am J Psychiatry. 2002;159(9):1548–1555.

18. Gulliver A, Griffiths KM, Christensen H. Perceived barriers and facilitators to mental health help-seeking in young people: a systematic review. BMC Psychiatry. 2010;10(1):113.
19. Ishikawa S, Okifuji A, Matsuoka H, Sakano Y. (2007). Cognitive Behaviour Therapy for Anxiety Disorders in Children and Adolescents: A Meta-Analysis. *Child and Adolescent Mental Health, 12*, 164-172.
20. James AC, James G, Cowdrey FA, Soler A, Choke A. (2013). Cognitive behavioural therapy for anxiety disorders in children and adolescents. *Cochrane database Syst Rev*. 6: CD004690.
21. Silverman WK, Pina AA, Viswesvaran C. (2008). Evidence-based psychosocial treatments for phobic and anxiety disorders in children and adolescents. *J Clin Child Adolesc Psychol, 37*, 105-130.
22. Reynolds S, Wilson C, Austin J, Hooper L. (2012). Effects of psychotherapy for anxiety in children and adolescents: A meta-analytic review. *Clinical Psychology Review, 32*, 251-262.
23. In-Albon T, Schneider S. Psychotherapy of childhood anxiety disorders: A meta-analysis. Psychother Psychosom 2007;76(1):15-24.
24. Cartwright-Hatton S, Roberts C, Chitsabesan P, Fothergill C, Harrington R. Systematic review of the efficacy of cognitive behaviour therapies for childhood and adolescent anxiety disorders. Br J Clin Psychol 2004 Nov;43(Pt 4):421-36.
25. DR1, den 30. september 2013, kl.21.30; *http://www.dr.dk/tv/se/tv-avisen/tv-avisen-788#!/05:20*, hentet den 09.10.13.
26. Kiropoulos, L. A., Klein, B., Austin, D. W., Gilson, K., Pier, C., Mitchell, J. & Ciechomski, L. (2008). Is internet-based CBT for panic disorder and agoraphobia as effective as face-to-face CBT? *Journal of Anxiety Disorders, 22*(8), 1273-1284.
27. Cunningham MJ, Wuthrich VM, Rapee RM, Lyneham HJ, Schniering CA, Hudson JL. The Cool Teens CD-ROM for anxiety disorders in adolescents : a pilot case series. Eur Child Adolesc Psychiatry. 2009;18(2):125–9.
28. Calear AL, Christensen H. Review of internet-based prevention and treatment programs for anxiety and depression in children and adolescents. Med J Aust. 2010;192(11 Suppl):S12–4.
29. Kendall, P. C., Khanna, M. S., Edson, A., Cummings, C., & Harris, M. S. (2011). Computers and psychosocial treatment for child anxiety: Recent advances and ongoing efforts. Depression and Anxiety, 28(1), 58-66. doi: http://dx.doi.org/10.1002/da.20757
30. Haug T, Nordgreen T, Ost LG, Havik OE. (2012). Self-help treatment of anxiety disorders: A meta-analysis and meta-regression of effects and potential moderators. *Clinical Psychology Review, 32*, 425-445.
31. Reger MA, Gahm GA. (2009). A meta-analysis of the effects of internet- and computer-based cognitive-behavioral treatments for anxiety. *J Clin Psychol, 65*, 53-75.
32. Andrews G, Cuijpers P, Craske MG, McEvoy P, Titov N. Computer therapy for the anxiety and depressive disorders is effective, acceptable and practical health care: a meta-analysis. PLoS One. 2010;5(10):e13196.
33. Haug T, Nordgreen T, Öst LG, Havik OE. Self-help treatment of anxiety disorders: a meta-analysis and meta-regression of effects and potential moderators. Clin Psychol Rev. 2012;32(5):425–45.
34. Andersson G, Cuijpers P, Carlbring P, Riper H, Hedman E. (In press). Internet-based vs. face-to-face cognitive behaviour therapy for psychiatric and somatic disorders: A systematic review and meta-analysis. World Psychiatry.
35. Khanna MS, Kendall PC. (2010). Computer-assisted cognitive behavioral therapy for child anxiety: Results of a randomized clinical trial. *J Consult Clin Psychol, 78*, 737-745.

36. March S, Spence SH, Donovan CL. (2009). The efficacy of an internet-based cognitive-behavioral therapy intervention for child anxiety disorders. *J Pediatr Psychol, 34*, 474-487.
37. Spence, S. H., Holmes, J. M., March, S., & Lipp, O. V. (2006). The feasibility and outcome of clinic plus Internet delivery of cognitive-behavior therapy for childhood anxiety. J Consult Clin Psychol, 74(3), 614-621. doi: http://dx.doi.org/10.1037/0022-006X.74.3.614
38. Spence SH, Donovan CL, March S, Gamble A, Anderson RE, Prosser S, Kenardy J. (2011). A randomized controlled trial of online versus clinic-based CBT for adolescent anxiety. *J Consult Clin Psychol, 79*, 629-642.
39. Wuthrich VM, Rapee RM, Cunningham MJ, Lyneham HJ, Hudson JL, Schniering CA. (2012). A Randomized Controlled Trial of the Cool Teens CD-ROM Computerized Program for Adolescent Anxiety. *Journal of the American Academy of Child and Adolescent Psychiatry, 51*, 261-270.
40. Tillfors M, Andersson G, Ekselius L, et al. A randomized trial of internet-delivered treatment for social anxiety disorder in high school students. Cogn Behav Ther. 2011;40(2):147–157.
41. Stallard, P., Richardson, T., Velleman, S., & Attwood, M. (2011). Computerized CBT (think, feel, do) for depression and anxiety in children and adolescents: Outcomes and feedback from a pilot randomized controlled trial. Behavioural and Cognitive Psychotherapy, 39(3), 273-284. doi: http://dx.doi.org/10.1017/S135246581000086X
42. Sethi S, Campbell AJ, Ellis LA. The use of computerized self-help packages to treat adolescent depression and anxiety. J Technol Hum Serv. 2010;28(3):144–160.
43. Stallard, P., Velleman, S. & Richardson, T. (2010). Computer use and attitudes towards computerized therapy amongst young people and parents attending child and adolescent mental health services. *Child and Adolescent Mental Health, 15*, 80-84.
44. James, A.M. (2007). Principles of youth participation in mental health services. *Medical Journal of Australia, 187*(suppl. 7), s57-s60.
45. Subrahmanyam, K. & Lin, G. (2007). Adolescents on the net: Internet use and well-being. *Adolescence, 42*, 659-677.
46. Livingstone, S., Haddon, L., Görzig, A., and Ólafsson, K. (2011). Risks and safety on the internet: The perspective of European children. Full Findings. LSE, London: EU Kids Online.
47. Arendt, K., Thastum, M., & Hougaard, E. (submitted). Efficacy of a Danish Version of the Cool Kids Program: A Randomized Wait-List Controlled Trial. Submitted to Behaviour Research and Therapy.
48. Gulliver, A., Griffiths, K. M., & Christensen, H. (2010). Perceived barriers and facilitators to mental health help-seeking in young people: A systematic review. BMC Psychiatry, 10. doi: http://dx.doi.org/10.1186/1471-244X-10-113
49. Kendall, P. C., Khanna, M. S., Edson, A., Cummings, C., & Harris, M. S. (2011). Computers and psychosocial treatment for child anxiety: Recent advances and ongoing efforts. Depression and Anxiety, 28(1), 58-66. doi: http://dx.doi.org/10.1002/da.20757
50. Richardson, T., Stallard, P., & Velleman, S. (2010). Computerised cognitive behavioural therapy for the prevention and treatment of depression and anxiety in children and adolescents: A systematic review. Clinical Child and Family Psychology Review, 13(3), 275-290. doi: http://dx.doi.org/10.1007/s10567-010-0069-9
51. Rickwood, D., & Bradford, S. (2012). The role of self-help in the treatment of mild anxiety disorders in young people: An evidence-based review. Psychology Research and Behavior Management, 5.

52. Rooksby, M., Elouafkaoui, P., Humphris, G., Clarkson, J., & Freeman, R. (2015). Internet-assisted delivery of cognitive behavioural therapy (CBT) for childhood anxiety: Systematic review and meta-analysis. Journal of Anxiety Disorders, 29(0), 83-92. doi: http://dx.doi.org/10.1016/j.janxdis.2014.11.006
53. Rapee, R., Lyneham, H., Schniering, C., Wuthrich, V., Abbott, M., Hudson, J. & Wignall, A. (2006). *Cool Kids "Chilled" Adolescent Anxiety Program.* Centre for Emotional Health, Macquarie University, Sydney.
54. Rapee RM, Lyneham HJ, Schniering CA, Wuthrich V, Abbott MJ, Hudson JL, Wignall A. (2010). *Cool Kids angstprogram for børn. Terapeutens manual.* Psykologisk Institut, Aarhus Universitet.
55. DSM-IV-TR. (2000). *Diagnostic and Statistical Manual of Mental Disorders, 4th ed. Text Revision.* Arlington, VA.: American Psychiatric Association.
56. Albano AM, Silverman WK. (1996). *Anxiety Disorders Interview Scedule for DSM - IV. Child version*. Oxford, Oxford University Press.
57. Lyneham HJ, Rapee RM. (2005). Agreement between telephone and in-person delivery of a structured interview for anxiety disorders in children. *Journal of the American Academy of Child and Adolescent Psychiatry, 44*, 274-282.
58. Cobham, V. E., Dadds, M. R., & Spence, S. H. (1999). Anxious children and their parents: What do they expect? Journal of Clinical Child Psychology, 28, 220–231. doi:10.1207/s15374424jccp2802_9
59. Arendt, K., Hougaard, E., & Thastum, M. (2014). Psychometric properties of the child and parent versions of Spence Children's Anxiety Scale in a Danish community and clinical sample. Journal of Anxiety Disorders, 28(8), 947-956. doi: http://dx.doi.org/10.1016/j.janxdis.2014.09.021
60. Nauta, M. H., Scholing, A., Rapee, R. M., Abbott, M., Spence, S. H., & Waters, A. (2004). A parent-report measure of children's anxiety: Psychometric properties and comparison with child-report in a clinic and normal sample. Behaviour Research and Therapy, 42(7), 813-839. doi: http://dx.doi.org/10.1016/S0005-7967(03)00200-6
61. Lyneham, H. J., Sburlati, E. S., Abbott, M. J., Rapee, R. M., Hudson, J. L., Tolin, D. F., & Carlson, S. E. (2013). Psychometric properties of the Child Anxiety Life Interference Scale (CALIS). Journal of Anxiety Disorders, 27(7), 711-719. doi: http://dx.doi.org/10.1016/j.janxdis.2013.09.008
62. Muris, P. (2001). A brief questionnaire for measuring self-efficacy in youths. Journal of Psychopathology and Behavioral Assessment, 23(3), 145-149. doi: http://dx.doi.org/10.1023/A:1010961119608
63. Muris, P. (2002). Relationships between self-efficacy and symptoms of anxiety disorders and depression in a normal adolescent sample. Personality and Individual Differences, 32(2), 337-348. doi: http://dx.doi.org/10.1016/S0191-8869(01)00027-7
64. Angold, A., Costello, E. J., Messer, S. C., & Pickles, A. (1995). Development of a short questionnaire for use in epidemiological studies of depression in children and adolescents. International Journal of Methods in Psychiatric Research, 5(4), 237-249.
65. Goodman, R., & Scott, S. (1999). Comparing the Strengths and Difficulties Questionnaire and the Child Behavior Checklist: Is small beautiful? J Abnorm Child Psychol, 27(1), 17-24. doi: http://dx.doi.org/10.1023/A:1022658222914
66. Niclasen, J., Teasdale, T. W., Andersen, A.-M. N., Skovgaard, A. M., Elberling, H., & Obel, C. (2012). Psychometric properties of the Danish Strength and Difficulties Questionnaire: The SDQ assessed for more than 70,000 raters in four different cohorts. PLoS One, 7(2). doi: http://dx.doi.org/10.1371/journal.pone.0032025

67. Horvath, A. O. (1981). An exploratory study of the working alliance: Its measurement and relationship to therapy outcome. Retrieved from http://search.proquest.com/docview/616700966? accountid=14468
68. Tracey, T. J., & Kokotovic, A. M. (1989). Factor structure of the Working Alliance Inventory. Psychological Assessment: A Journal of Consulting and Clinical Psychology, 1(3), 207-210. doi: http://dx.doi.org/10.1037/1040-3590.1.3.207
69. Topp, C. W., Østergaard, S. D., Søndergaard, S., & Bech, P. (2015). The WHO-5 Well-Being Index: A Systematic Review of the Literature. Psychotherapy and Psychosomatics, 84(3), 167-176.

Appendix 1: Project measures

- ADIS-IV C/P administered over telephone (pre, post, follow-up) A/P
- Demographic questions (adolescent age and gender, family constellation, school type, average days of school absence, previous professional contact regarding child anxiety, medication history, parent age, marital status, number of children, name and level of highest education, occupation, household income) (pre) P
- Spence Child Anxiety Scale (SCAS; 2,59,60) (pre, post, follow-up) A/P
- Children's Anxiety Life Inference Scale (CALIS; 61) (pre, post, follow-up) A/P
- Self-Efficacy Questionnaire for Children (SEQ-C; 62, 63) (pre, post) A
- Short Moods and Feelings Questionnaire (SMFQ-c/p; 64) (pre, post) A/P
- WHO-5 Well-being Index Questionnaire (WHO-5; 69) (pre, post, follow-up) A
- Strength and Difficulties Questionnaire for Youth (SDQ; 65, 66) (pre, post) A/P
- Working Alliance Inventory – Short form (WAI-S; 67; 68) (after week 4 and 8, post) A
- Experience of Service Questionnaire (ESQ; Commission for Health Improvement, 2002) (post) A/P
- Adherence, i.e. degree of program completion, number of logins to the program web site (to be monitored in the program back end) (post) A

Further questions

- How du you feel about going to therapy soon? (pre) A
- How do you feel about your treatment being delivered online? (pre) A
- How comfortable do you feel using the computer and internet? (pre) A
  Not at all   Some   Fairly   Very
- How far have you made it in the program? (post) A
- On average, how much time have you spend weekly working with the online modules? (post) A
  No time   10-30 min   30-60 min.   1-2 hours   2-5 hours   5-10 hours   More than 10 hours
- On average, how much time have you spend weekly doing program relevant exercises away from the computer? (post) A
  No time   10-30 min   30-60 min.   1-2 hours   2-5 hours   5-10 hours   More than 10 hours
- On average, how much time have you spend weekly helping your child with program relevant activities? (post) P
  No time   10-30 min   30-60 min.   1-2 hours   2-5 hours   5-10 hours   More than 10 hours

Pre = before treatment start
Post = 15 weeks after treatment start
Follow-up = 3 months after treatment completion
A = completed by the adolescent
P = completed by the parents
