# Supplementary material for: A randomized controlled trial examining the efficacy of an internet-based cognitive behavioral therapy program for adolescents with anxiety disorders
Source: PLoS One. 2019 Sep 18;14(9):e0222485. doi: 10.1371/journal.pone.0222485 (PMC6750608; doi:10.1371/journal.pone.0222485)
Supplement: S3 File — (PDF) [file pone.0222485.s003.pdf]

Forsøgsprotokol

En randomiseret kontrolleret undersøgelse af internetbaseret behandling af unge med angstlidelser

Baggrund

Angst er en af de mest udbredte psykiske lidelser blandt børn og unge (1,2); forskning viser, at 2,5 – 5 % af børn i vesten har en angstlidelse, mens livstidsprævalensen anslås at være mellem 15 og 20 % (1,3-5). Tilstedeværelsen af en angstlidelse medfører betydelige negative konsekvenser for det enkelte individ, herunder problemer i skolen, med vennerne og i familien (6,7), og mange angstlidelser hos børn og unge har et kronisk forløb som bevirker, at angsten varer ved op gennem ungdomsårene til voksenlivet, hvis ikke den behandles (2,8,9,10). Endvidere kan tilstedeværelsen af angst tidligt i livet være markør for udviklingen af andre psykiske lidelser senere hen som depression og misbrugsproblemer (4,11,12). Således bliver angstlidelserne et bekosteligt samfundsproblem (13). Undersøgelser tyder dog på, at kun en mindre procentdel af børn og unge med angstlidelser modtager behandling (14,15,16,17). Foruden et generelt begrænset udbud af behandlinger til børn og unge kan manglen på behandling muligvis også skyldes modvilje mod at søge hjælp grundet frygt for stigmatisering, utryghed eller ubehag forbundet med det at skulle påtale psykiske vanskeligheder samt en mulig præference blandt især de unge for selvhjælp (18).

Kognitiv adfærdsterapi (KAT) er en veldokumenteret og effektiv behandlingsform til børn og unge med angstlidelser (19-24). Den seneste og størst anlagte metaanalyse (22) af 55 randomiserede kontrollerede studier af KAT brugt til behandlingen af børn og unge (alder: 6 til 19) med angstlidelser (n = 4256) rapporterer om en gennemsnitlig effektstørrelse på d = 0.77 sammenlignet med venteliste-kontrolgrupper, og en effektstørrelse på d = 0.39 ved sammenligningen af KAT med aktive kontrolgrupper (fx opmærksomheds-placebo, afslapningstræning eller placebomedicinering).

På internationalt plan har man de seneste år kunnet se en stigning i udviklingen og brugen af internetbaserede kognitive adfærdsterapeutiske programmer (IKAT), blandt andet som en måde at reducere udgifterne og øge tilgængeligheden til psykologisk behandling af angstlidelser (25,26). Brugen af internet og computer / tablets / smartphones har desuden et stort potentiale i forhold til at overkomme nogle af før nævnte barrierer for terapeutisk behandling ved blandt andet at tilbyde brugerne større anonymitet, lettere tilgængelighed i forhold til både tid og sted for behandlingen, større fleksibilitet med hensyn til behandlingens tempo og specifikke indsatsområder samt muligt større appel til børn og unge ved brugen af interaktive og stimulerende programelementer (27,28,29).

IKAT har vist sig at være en effektiv behandlingsform overfor voksne med angstlidelser; effekten svarer til den effekt der opnås ved terapeutadministreret KAT (30-34). Der findes dog fortsat relativt få randomiserede kontrollerede undersøgelser af IKAT til børn og unge med angstlidelser; pt. kan der lokaliseres hhv. tre RCT studier af IKAT til børn (7-14 år) (35,36,37), tre RCT studier af IKAT til unge (12-21 år) (38,39,40) og to RCT studier af IKAT til både angst og depression blandt unge (11-25 år) (41,42). I samtlige studier er IKAT fundet ventelistekontrollerne overlegen og nær ved lige så effektiv som terapeutadministreret KAT. Studierne er geografisk set lokaliseret i USA (35), Australien (36-39,42), England (41) og Sverige (40). Til dato er intet IKAT-program for børn eller unge med angstlidelser blevet udviklet eller evalueret i Danmark.

Internetbaseret terapi antages at være særligt passende for unge: 1) fordi terapien kan tilgås til enhver tid og således er fleksibel i forhold til den unges øvrige forpligtelser og aktiviteter; 2) fordi internettet tilbyder en mere privat, anonym og fortrolig sfære omkring terapien sammenlignet med almindelig face-to-face behandling; en sfære som særligt værdsættes af unge i teenagealderen (43,44); 3) fordi internettet er et medie, som de fleste unge er komfortable og trygge ved at anvende (45). Ifølge en nylig rapport fra EU Kids Online (46), baseret på mere end 25.000 børn (9-16 år) og deres forældre fra 25 europæiske lande, heriblandt Danmark, er brug af internettet nu en integreret del af børnenes hverdag; 93 % af de adspurgte børn og unge tilgår internettet mindst én gang om ugen – for 80 % af de 15-16 årige sker det næsten dagligt. Børnene bruger i gennemsnitligt 88 minutter om dagen på aktiviteter 'online'. Det er derfor ikke utænkeligt, at et internetbaseret behandlingsprogram til unge også i Danmark gemmer på et stort potentiale.

Formål

Projektets formål er, i en randomiseret kontrolleret undersøgelse, at afprøve anvendeligheden og evaluere effekten af en dansk udgave af et nyligt udviklet australsk IKAT-program, 'Chilled Out', til behandling af unge mellem 13 og 17 år med angstlidelser sammenlignet med en venteliste kontrolgruppe.

Undersøgelsen sker som en videreudvikling af et tidligere forskningsprojekt, 'Evidensbaseret behandling af børn og unge med angstlidelser', ved Angstklinikken for børn og unge, Psykologisk Institut, Aarhus Universitet (projekt-id M-20110019), hvor man undersøgte effekten af en dansk version af det manualiserede gruppebehandlingsprogram 'Cool Kids' med gode resultater (48,2 % var fri af deres angstdiagnoser efter behandlingens afslutning sammenlignet med 5,7 % i venteliste kontrolgruppen) (47).

Projektets hypoteser

Chilled Out programmet forventes at passe ind i de unges vaner og almene levevis og derfor at blive godt modtaget af de unge deltagere.

Programmet forventes, på baggrund af eksisterende reviews og meta-analyser af lignede IKAT-programmer til børn og unge (48-52), at have en signifikant bedrende effekt på de unges angst (målt ved ADIS, SCAS og CALIS; se uddybning nedenfor) samt på deres generelle mentale helbred (målt ved SMFQ-c/p, Quality of life og SDQ; se uddybning nedenfor) sammenlignet med venteliste kontrolgruppen.

Ved benchmarking analyser forventes Chilled Out at vise sig nær ved lige så effektiv som sammenlignelige terapeutadministrerede gruppebehandlingsprogrammer (53,54).

Effekten af internetinterventionen forventes vedligeholdt ved tre måneders opfølgning.

Deltagernes specifikke diagnoser (målt ved ADIS) og alder, kvaliteten af den terapeutiske alliance (målt ved WAI-S), deltagernes grad af selvtillid (målt ved SEQ-C) samt graden af forældreinvolvering (målt ved gennemsnitligt antal timer om ugen brugt på behandlingsrelevante aktiviteter) forventes delvist at moderere eller mediere behandlingseffekten.

Metode

Design

Studiet er en randomiseret kontrolleret undersøgelse af 70 unge fordelt i to grupper:

1) IKAT (n = 35)

2) venteliste kontrol (n = 35)

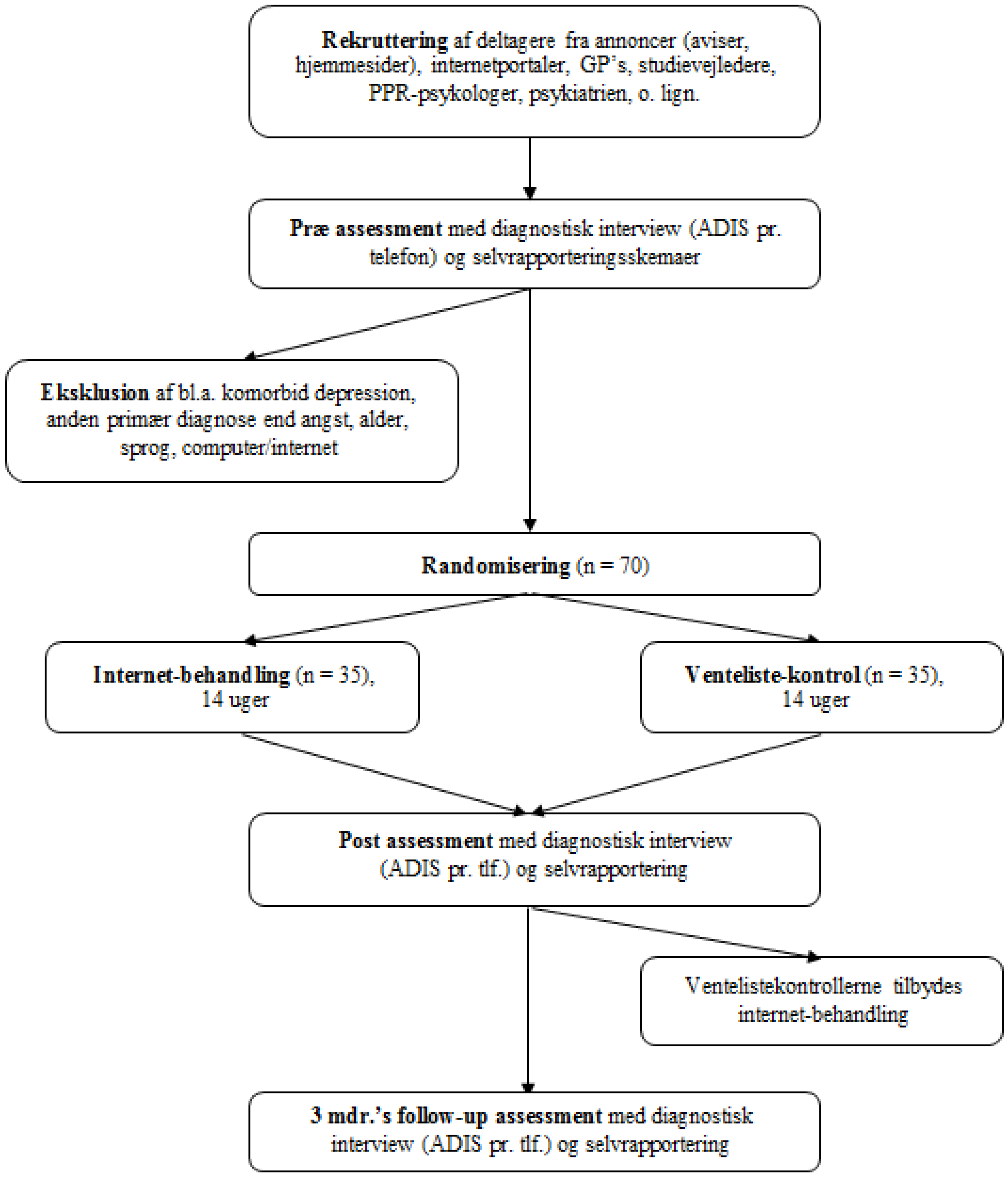

Deltagere
De 70 deltagere vil blive rekrutteret fra Angstklinikkens venteliste, samt ved annoncering i Angstklinikkens nyhedsbrev, på klinikkens hjemmeside, på Aarhus Universitets hjemmeside, på Angstforeningens hjemmeside, i diverse dagblade, på udvalgte Facebook-sider (opslag på hhv. Silke Stjerneklars og Mikael Thastums personlige sider samt på siderne 'Børn med angst – forum for forældre', 'Angstforeningen', 'Danske psykologer'), via studievejledere og PPR-psykologer rundt om i landet samt ved henvisninger fra professionelle (se 'Rekutteringstekst', appendiks IV). Rekrutteringsforløbet påregnes at foregå i perioden 1. juni til 1. oktober, 2015.

Generelt demonstreres der i litteraturen store effektstørrelser ved sammenligninger mellem en aktiv behandling og en kontrolgruppe uden intervention (36). Styrkeberegninger, baseret på t-test af forskellen mellem to uafhængige gennemsnit samt tidligere studier af IKAT programmer til børn og unge med angstlidelser har vist, at en total sample størrelse på 52 vil give en styrke på .80 til at kunne opfange en stor effektstørrelse (d = .80) ved α = .05. En sample størrelse på 70 antages derfor at kunne opfange en klinisk meningsfuld forskel imellem de to grupper (behandling versus ingen behandling) samt at kunne rumme et forventet frafald på mellem 2-13 % (38,39,40).

In- og eksklusionskriterier
Inkluderet i projektet bliver 70 unge mellem 13 og 17 år diagnosticeret med angst (separationsangst, generaliseret angst, social fobi, specifik fobi, OCD og panikangst) som primær diagnose i overensstemmelse med DSM-IV (2000) (55). Deltagerne skal desuden have direkte adgang til en computer med stabilt internet i hjemmet samt være i stand til at læse og skrive på dansk. Eksklusionskriterierne er som følger: PTSD som primær diagnose, høj grad af komorbid depression (CSR $\geq$6, målt ved ADIS-IV-c/p (56)), alkohol eller stofmisbrug, aktuel suicidal eller alvorlig selvskadende adfærd, gennemgribende udviklingsforstyrrelse eller signifikant indlæringsforstyrrelse, dårlig begavelse samt psykotiske symptomer. Deltagerne opfordres desuden til ikke at lave ændringer i deres eventuelle medicinstatus, mens undersøgelsen står på.

Procedure
Deltagere på ventelisten kontaktes telefonisk af en studerende fra Angstklinikken. Her aftales dato og tidspunkt for det kommende diagnostiske interview.

Umiddelbart herefter sendes indkaldelsesbrev til familien (appendiks V), hvori aftalte dato og tidspunkt for den diagnostisk samtale bekræftes, de nærmere omstændigheder for interviewet uddybes og familien informeres om proceduren fremadrettet. Se nærmere beskrivelse af forløb for indhentelse af informeret samtykke nedenfor.

Kort herefter vil den unge og forældrene (både mor og far) modtage en elektronisk spørgeskemapakke (appendiks V), som de bliver bedt om at udfylde hurtigst muligt. Hhv. én og to uger herefter sendes ved manglende svar en påmindelsesmail. Er skemaerne fortsat ikke besvaret tre uger efter afsendelse, vil familien blive kontaktet telefonisk af den ansvarlige terapeut.

Det diagnostiske interview vil blive foretaget med 'the Anxiety Disorder Interview Schedule for DSM-IV, Child version / Parent version' (ADIS-IV c/p) (56) særskilt med både den unge og den ene forælder over telefon, hvilket er vist at have sammenlignelig validitet med face-to-face administreringen samt høj interrater reliabilitet (57,58). De kliniske vurderinger udføres af kandidatstuderende ved Psykologisk Institut, træ-

nede i brugen af ADIS-IV c/p, under regelmæssig supervision af uddannede psykologer. Alle samtaler optages med det formål efterfølgende at vurdere diagnosernes reliabilitet ved samrating (20 %).

Efter den initiale vurdering vil de inkluderede deltagere i blokke af 10 (hhv. 5/5) blive randomiseret til enten IKAT-intervention ’Chilled Out’ eller kontrolbetingelsen (14 ugers venteliste). Randomiseringen vil blive udført af en computeralgoritme, overvåget og assisteret af en sekretær uden tilknytning til Angstklinikken. Kontroldeltagerne vil blive tilbudt behandling med Chilled Out programmet efter ventelisteperioden er afsluttet. Alle deltagere vil have adgang til programmet minimum 3 mdr. efter behandlingsafslutning.

Familien vil få besked på, om de er inkluderede i undersøgelsen eller ej samt hvilken af de to grupper (intervention eller venteliste), de er lodtrukket til, indenfor en uge efter det diagnostiske interview. Unge i interventionsgruppen modtager umiddelbart herefter et brev (’Programinformation’, appendiks V) med hjemmesidens internetadresse, et personligt brugernavn og en midlertidig adgangskode, en specifik opstartsdato fra hvilken det vil være muligt for den unge at logge ind i programmet samt en oversigt med dato og tidspunkt for behandlingsforløbets kommende 14 ugers opkald. Dette brev vil ligeledes indeholde en kort præsentation af den unges terapeut samt terapeutens mailadresse.

Deltagernes brugernavne udgøres af den unges for- og efternavn. Den midlertidige adgangskode genereres med en online adgangskodegenerator (*Norton Identity Safe Password-generator)*. Deltagernes brugerprofiler (herunder navn, fødselsdato, mailadresse, køn, profilens udløbsdato, brugernavn og midlertidige adgangskode) indtastes manuelt i programmets back end af programadministratoren. Første gang en deltager logger ind i programmet, vil han eller hun automatisk blive bedt om at ændre sin adgangskode. På denne måde sikres det, at kun den unge selv er bekendt med sine legitimationsoplysninger. Den unges terapeut samt programadministratoren vil, som de eneste ud over den unge selv, kunne tilgå den unges programflade (dvs. have adgang til udfyldte arbejdsark, en oversigt over den unges brug af programmet, graf over den unges fremgang o.lign.), via en funktion i programmets backend kaldet ’impersonating’. Dette vil den unge blive informeret om af terapeuten ved første samtale.

Behandlingen

Det internetbaserede behandlingsprogram Chilled Out er oprindeligt udviklet på Centre for Emotional Health, the Psychology Department at Macquarie University, Australien, som en videreudvikling af det manualiserede gruppebehandlingsprogram ’Chilled Adolescents Anxiety Program’ for 13 til 17-årige (54,59), oprindeligt skabt af prof. Ron Rapee og kollegaer. Angstklinikkens leder, prof. Mikael Thastum, har fået tilladelse til at oversætte, tilpasse og anvende internetprogrammet som led i et kontinuerligt forskningssamarbejde med Rapee. Chilled Out programmet er udviklet til brug på den unges hjemmecomputer under kontinuerlig støtte og vejledning fra den unges forældre. Programmets bagvedliggende teori er baseret på den kognitive adfærdsterapeutiske færdighedstræningstilgang og omfatter komponenter som kognitiv omstrukturering, gradueret eksponering, responshæmning, adfærdseksperimenter, problemløsning, opmærksomhedstræning samt udviklingen af assertiv adfærd.

Chilled Out programmet består af i alt otte online færdighedstræningsmoduler af hver cirka 35 minutters varighed, som gennemføres over en periode på 12-14 uger. Ved hjælp af interaktive eksempler, øvelser og informationsvideoer leveret via en kombination af forskellige medier (tekst, lyd, illustrationer, tegnefilm og live-video) lærer den unge en række kognitive adfærdsterapeutiske strategier, og udstyres således med evnen til på egen hånd at håndtere, reducere og kontrollere sin angst.

Overskrifterne i programmets otte moduler er som følger:

1. At forstå angst: introduktion til programmets funktioner samt psykoedukation om angst
2. Målsætning: guider den unge til at sætte sig mål for behandlingsforløbet, introducerer belønning/selvbelønning samt forventningsafstemning ift. det forestående behandlingsforløb
3. Realistisk tænkning I: fokuserer på sammenhængen mellem tanker og følelser og introducerer realistisk tænkning samt bevisførelse
4. Trappestiger I: at stå ansigt til ansigt med frygten; angstbekæmpelse via opbygning og planlægning af trappestiger
5. Trappestiger II: gennemgår trappestigerevidering og adfærdseksperimenter
6. Realistisk tænkning II: indre realistisk tænkning, 'laden som om' samt opmærksomhedstræning, her kaldet bekymringssurfing
7. Andre færdigheder: præsenterer problemløsning, feedback fra andre, assertiv kommunikation og adfærd samt afslapning
8. Stadig chilled: opsummering og tilbagefaldsforebyggelse

Indlejret i programmets brugerflade er desuden en række redskaber og features, som den unge kan tilgå via menupunkter øverst på skærmen. Her findes blandt andet den unges profiloplysninger, oversigt over den unges brug af programmet (fx antal log ind), en grafisk afbildning af den unges fremgang udformet på baggrund af et programindlejret ugentligt spørgeskema (se appendiks V), en dagbog, som den unge kan skrive personlige notater i, arbejdsark til alle øvelser i programmet, en beskedfunktion hvorigennem den unge kan få support til programmets tekniske aspekter, akuthjælp med råd og telefonnumre til forskellige telefoniske hjælpelinjer samt generelle beskrivelser af hjemmesidens vilkår og betingelser.

I tillæg til internetprogrammet modtager den unges forældre et hæfte, som introducerer de kognitive adfærdsterapeutiske kernefærdigheder, den unge lærer, og drøfter måder, hvorpå forældrene kan hjælpe deres barn med anvendelsen af de tillærte færdigheder. Forældrene opfordres til løbende at samtale med den unge om programmet samt til at assistere den unge i gennemførelsen af praktiske opgaver i de situationer, hvor det synes relevant. Endvidere modtager forældrene en invitation til at deltage i en lukket gruppe på facebook ('Forældre til unge i Chilled Out'), hvor de har mulighed for at rejse spørgsmål, give gode råd, udveksle erfaringer o. lign. med andre forældre, hvis børn tillige er inkluderet i internetbehandlingsforløbet (se appendiks V). Gruppens aktiviteter vil være under kontinuerligt tilsyn af den projektansvarlige for at sikre en god tone og et relevant indhold i de opslåede indlæg. I tilfælde, hvor dette vurderes ikke at være opfyldt, vil den eller de relevante forældre blive kontaktet af projektansvarlige og instrueret i gruppens formål og retningslinjer (se appendiks V). Forældrene vil med invitationen også modtage en samtykkeerklæring (se appendiks III), hvori de giver tilladelse til, at alle eller dele af informationsudvekslingerne i Facebookgruppen må anvendes i anonymiseret form i forsknings- og publiceringshenseender. Det vil være en forudsætning for inklusion af forældre i gruppen, at de har indsendt den underskrevne samtykkeerklæring i en vedlagt svarkuvert. Deltagelse i gruppen er frivillig og vil ikke få indflydelse på det behandlingsforløb, som familien modtager fra klinikken.

Ugentligt vil den unge desuden modtage et støttende telefonopkald af kortere varighed foretaget af den tilknyttede terapeut. Opkaldets formål er at tjekke den unges og forældrenes forståelse af programmet, at assistere indlæringen og anvendelsen af programmets færdigheder i den unges liv samt at støtte problemløsningen af de eventuelle vanskeligheder, som familierne måtte støde på undervejs. De terapeutiske opkald vil blive udført af kandidatstuderende ved Psykologisk Institut, trænede i kognitiv adfærdsterapi og

under regelmæssig supervision af uddannede psykologer. Hver terapeut udstyres desuden med en detaljeret skriftlig terapeutmanual, udviklet på baggrund af en nylig pilotafprøvning af programmet. Telefonopkaldene vil blive optaget og vurderet med henblik på at afklare samtalernes primære indhold, de fornødne terapeutkompetencer samt deltagernes adherence.

Foruden de ugentlige opkald vil deltagerne have mulighed for at kontakte den tilknyttede terapeut via mail, i fald der opstår spørgsmål til eller vanskeligheder i forbindelse med den unges gennemførsel af programmet. Deltagerne kan forvente svar på mail indenfor to hverdage. Mailkorrespondancens ikke-akutte funktion vil blive understreget.

Tre måneder efter behandlingsafslutningen vil deltagerne blive ringet op af den tilknyttede terapeut til en såkaldt 'booster-samtale'. Formålet med denne samtale er at støtte den unges fortsatte arbejde med de tillærte færdigheder samt at problemløse eventuelle vanskeligheder, som familierne måtte være stødt på siden behandlingens afslutning. Desuden vil der, på baggrund af den unges fremgang og generelle velbefindende, her blive foretaget en vurdering af, hvorvidt den unges adgang til Chilled Out programmet skal afvikles eller forlænges yderligere tre måneder. Tidsmæssigt ubegrænset adgang er ikke muligt indenfor rammerne af nærværende projekt, eftersom enhver deltager med adgang til programmet også bør have adgang til programrelevant support. En sådan support kan af projektgruppen kun stilles til rådighed indenfor tidsmæssigt og økonomisk begrænsede rammer.

Målemetoder og data
Projektet anvender en række selvrapporteringsskemaer, svarende til sådanne der indgår i Angstklinikkens fortløbende forskning (se bilag 1, s. 15). De relevante spørgeskemaer udsendes via mail præ, efter 4. og 8. uge, post behandlingen, tre måneder samt et år efter behandlingsafslutningen og udfyldes af både unge og forældre på familiernes hjemmecomputere. Alle data administreres elektronisk ved brug af computerprogrammet SurveyXact (http://www.surveyxact.dk) og importeres direkte til statistikprogrammet SPSS, hvor de opbevares på et låst drev. De kliniske ratings vil blive administreret og udført af kliniske psykologer i samarbejde med tilknyttede forskningsassistenter.

Opbevaring af data
Alle oplysninger vedrørende deltagerne beskyttes efter lov om behandling af personoplysninger og lov om patienters retstilling. Data (herunder patientjournaler, brugernavne og adgangskoder til programmet) vil blive opbevaret på Angstklinikken, Psykologisk Institut, Aarhus Universitet, under ansvar af Silke Stjerneklar og Mikael Thastum.

Statistiske analyser
Der anvendes 'intention to treat' procedure ved de statistiske analyser; alle drop outs vil således få tilsendt projektets spørgeskemapakke ved både midt, post og followup samt blive indkaldt til ADIS-telefoninterview ved post og followup.

Drop-out analyser: sociodemografisk data, diagnoser, behandlingsrelaterede og psykosociale data fra deltagere og drop-outs vil blive sammenholdt og evalueret i forhold til resultaternes generaliserbarhed. Senere analyser vil tage højde for eventuelt fundne forskelle.

Baseline sammenligning af grupperne: en indledende sammenligning af data fra de to grupper (hhv. interventionsgruppen og venteliste kontrolgruppen) vil blive foretaget ved baseline med brug af t-tests og chi^2 tests med det formål at evaluere resultatet af randomiseringen. Senere analyser vil blive statistisk tilpasset i fald der forefindes signifikante gruppeforskelle.

Primære effekter: hovedeffekten af behandlingen målt ved de angivne outcome variabler (bilag 1) vil blive statistisk vurderet ved brug af mixed-model repeated-measures ANOVA's (Gruppe x Tid). Hvis der forefindes statistisk signifikante gruppe x tid interaktioner, vil data blive analyseret yderligere med post-hoc-tests/planned contrast analyser.
Undersøgelsesresultaterne vil desuden blive benchmarked med tilsvarende undersøgelser og sammenlignet med resultater fra Angstklinikkens tidligere venteliste kontrolstudie.

Etiske overvejelser

Der vurderes ikke at være etiske problemer i forbindelse med undersøgelsen. Tilladelse og godkendelse fra De Videnskabsetiske Komitéer – Region Midtjylland vil blive indhentet i henhold til gældende love og regler. Projektet anmeldes desuden til datatilsynet.

Projektgruppen er opmærksom på de etiske aspekter, der kan være til stede ved inddragelsen af mindreårige i sundhedsvidenskabelige forsøg. Inddragelsen af de 13 til 17-årige i nærværende projekt vurderes bydende nødvendig for projektets gennemførelse, da det netop er denne aldersgruppe behandlingsprogrammet Chilled Out er udviklet til. Projektet forventes at kunne bidrage til en væsentlig mindskelse af deltagernes psykiske vanskeligheder, herunder i særdeleshed deres angstsymptomer, og indebærer minimale risici og gener for de unge. Chilled Out programmet forventes at passe godt ind i de unges vaner og almene levevis (jf. indledende argumentation) og derfor at blive taget vel imod af forsøgspersonerne. Jf. gældende lovgivning udvikles særskilt deltagerinformation til de 15 til 17-årige (appendiks II).

Deltagelse i undersøgelsen er frivillig, og familierne kan til enhver tid afbryde deres deltagelse, uden at det får betydning for nogen anden behandling, familien måtte være interesseret i at opsøge udenfor Angstklinikken. Da klinikken ikke har behandlingsforpligtelse, vil familier, der takker nej til at indgå i projektet, ikke kunne blive tilbudt anden behandling i klinikkens regi. De deltagende familier informeres både skriftligt og mundtligt om undersøgelsens formål og metoder og underskriver samtykkeerklæring inden interventionens start.

Deltagerne vil ikke modtage nogen form for økonomisk kompensation for at deltage i undersøgelsen.

Brugen af kognitiv adfærdsterapi er et anerkendt terapeutisk redskab, også når det tilbydes via internettet, og der er ingen kendte negative bivirkninger, risici eller ulemper forbundet med terapiformen.

Brugen af en venteliste kontrolgruppe sikrer, at projektet opnår forskningsmæssigt vigtig kontroldata. Unge, der randomiseres til ventelistekontrol gruppen, vil modtage internetbehandlingen efter en 14 ugers venteperiode.

Lov om behandling af personoplysninger vil blive overholdt.

Resultater fra undersøgelsen, være de positive, negative eller inkonklusive, vil blive offentliggjort i internationale tidsskrifter. Enkelte deltagere vil ikke kunne identificeres i offentliggjort data.

Forløb for indhentelse af informeret samtykke

Den første kontakt til potentielle projektdeltagere foregår enten via klinikkens hjemmeside (www.psy.au.dk/angstklinik) eller via annoncetekster ('Rekrutteringstekst', appendiks IV) i diverse medier. Familier, som på denne baggrund henvender sig til klinikken, anmodes om at sende en mail med en kort beskrivelse af deres barns vanskeligheder. Hvis det herudfra skønnes, at barnet kunne opfylde projektets inklusionskriterier, sendes en mail til familien med et tilbud om at komme på klinikkens venteliste. Klinikkens folder vedlægges denne mail (se 'Angstklinikkens velkomstfolder', appendiks IV). Folderen indeholder bl.a. information om, at familien, hvis de tilbydes behandling, deltager i et lodtrækningsforsøg med ventelistekontrol, og at nogle familier således må vente i 14 uger inden de kan påbegynde behandlingen. Ved positiv tilbagemelding på denne mail skrives familien op på ventelisten.

I perioden fra den 1. august til den 31. december (indenfor hvilken de diagnostiske interviews kommer til at foregå) vil familier på ventelisten blive kontaktet telefonisk af en studerende fra Angstklinikken. Her aftales dato og tidspunkt for det kommende diagnostiske interview. Ved denne samtale informeres forældrene og den unge ligeledes mundtligt om projektet. Den studerende, som foretager opkaldet, er sidst på studiet, under supervision og med erfaring i formidling af informationer til børn og unge. Den fornødne information vil blive givet på hensynsfuld vis, tilpasset hhv. forældrenes og den unges individuelle forudsætninger for at forstå og tage stilling hertil.

Projektgruppen er opmærksom på, at mundtlig information vedrørende deltagelse i forskningsforsøg som udgangspunkt bør gives ansigt til ansigt. Når vi alligevel vælger alene at give den mundtlige information telefonisk beror det på selve undersøgelsens rationale, den *internetbaserede interventionsform*. Et af hovedargumenterne for denne behandlingsmetode er netop, at man fritager patienterne for flere af de tidsmæssige og økonomiske negative aspekter, der gør sig gældende ved almindelig 'face-to-face' behandling. Vi ønsker, at nærværende undersøgelse kan repræsentere et bud på en *landsdækkende, let tilgængelig, økonomisk besparende indsats* for både patienter og behandlingssystem - et mål, som vi ikke i nær samme grad kan opnå, hvis deltagerne skal møde op på klinikken til et informationsmøde. Vi er bekymrede for, at et sådant påkrævet møde kan udelukke familier, som ikke har råd til eller mulighed for at rejse afstanden til klinikken – familier, som i dag også udelukkes fra de få behandlingstilbud, der eksisterer rundt om i landet. Skulle enkelte deltagere, unge eller forældre, give udtryk for, at de ønsker et personligt informationsmøde, vil vi naturligvis imødekomme forespørgslen og istandsætte et sådant på klinikken.

Umiddelbart efter at familien har fået den mundtlige information sendes indkaldelsesbrev til familien (appendiks V), hvori aftalte dato og tidspunkt for den diagnostisk samtale bekræftes, de nærmere omstændigheder for interviewet uddybes og familien informeres om proceduren fremadrettet. Vedlagt i dette brev findes også stedfortrædende samtykkeerklæring (ved fælles forældremyndighed indhentes fra begge forældre), samtykkeerklæringer vedrørende hhv. publikationer, lydoptagelser og informationsudveksling (appendiks III), skriftlig deltagerinformation (appendiks II), notat om forsøgspersonens rettigheder (appendiks V) samt en frankeret svarkuvert. Deltagerne bedes returnere de underskrevne erklæringer indenfor tre hverdage; ved manglende svar ringes familien op af klinikkens sekretær med en venlig påmindelse.

Projektledelse og økonomisk støtte

Projektet udføres fra Angstklinikken for Børn og Unge, Psykologisk Institut, School of Business and Social Sciences, Aarhus Universitet. Projektet er udviklet i et samarbejde mellem Silke Stjerneklar, Cand.psych., Ph.d. studerende og projektansvarlig; Mikael Thastum, Professor, Ph.d., Cand.psych. og leder af Angstklinikken; samt Esben Hougaard, Professor og Cand.psych.

Løn til projektansvarlige er finansieret gennem en ph.d. stipendiat fra den Samfundsvidenskabelige ph.d. skole, Aarhus Universitet. Stipendiaten er gældende i perioden 1. februar 2014 til 31. januar 2017. De resterende udgifter til projektet, herunder løn til klinisk psykolog og sekretær på klinikken, dækkes af en projektbevilling modtaget fra Trygfonden på kr. 2,5.000.000.

Referenceliste

1. Rapee, R.M., Schniering, C.A. & Hudson, J.L. (2009). Anxiety disorders during childhood and adolescence: Origins and treatment. *Annual Review of Clinical Psychology, 5*, 311-341.
2. Spence, S.H. (1998). A measure of anxiety symptoms among children. *Behavior Research and Therapy, 36*(5), 545-566.
3. Costello EJ, Mustillo S, Erkanli A, Keeler G, Angold A. (2003). Prevalence and development of psychiatric disorders in childhood and adolescence. *Arch Gen Psychiatry, 60*, 837-844.
4. Beesdo K, Knappe S, Pine DS. (2009). Anxiety and anxiety disorders in children and adolescents: Developmental issues and implications for DSM-V. *Psychiatr Clin North Am, 32*, 483-524.
5. Costello EJ, Egger HL, Copeland W, Erkanli A, Angold A. (2011). The developmental epidemiology of anxiety disorders: Phenomenology, prevalence, and comorbidity. In: *Anxiety disorders in children and adolescents. 2 ed.* Silverman WK, Field AP, Eds. Cambridge, Cambridge University Press, 56-75.
6. Davis TE, Ollendick TH, Nebel-Schwalm M. (2008). Intellectual ability and achievement in anxiety-disordered children: A clarification and extension of the literature. *Journal of Psychopathology and Behavioral Assessment, 30*, 43-51.
7. Angold A, Messer SC, Stangl D, Farmer EM, Costello EJ, Burns BJ. Perceived parental burden and service use for child and adolescent psychiatric disorders. Am J Public Health. 1998;88(1):75–80.
8. Kessler RC, Berglund P, Demler O, Jin R, Merikangas KR, Walters EE. (2005). Lifetime prevalence and age-of-onset distributions of DSM-IV disorders in the National Comorbidity Survey Replication. *Arch Gen Psychiatry, 62*, 593-602.
9. Muris, P. (2006). The pathogenesis of childhood anxiety disorders: Considerations from a developmental psychopathology perspective. *International Journal of Behavioral Development, 30*(1), 5-11.
10. Woodward LJ, Fergusson DM. Life course outcomes of young people with anxiety disorders in adolescence. J Am Acad Child Adolesc Psychiatry. 2001;40(9):1086–93.
11. Bittner A, Egger HL, Erkanli A, Jane CE, Foley DL, Angold A. What do childhood anxiety disorders predict? J Child Psychol Psychiatry 2007 Dec;48(12):1174-83.
12. Kovacs M, Gatsonis C, Paulauskas SL, Richards C. Depressive disorders in childhood. IV. A longitudinal study of comorbidity with and risk for anxiety disorders. Arch Gen Psychiatry 1989 Sep;46(9):776-82.
13. Bodden DH, Dirksen CD, Bogels SM. (2008). Societal burden of clinically anxious youth referred for treatment: A cost-of-illness study. *J Abnorm Child Psychol, 36*, 487-497.
14. Essau CA, Conradt J, Petermann F. (2000). Frequency, comorbidity, and psychosocial impairment of anxiety disorders in German adolescents. *J Anxiety Disord, 14*, 263-279.
15. Essau CA. Frequency and patterns of mental health services utilization among adolescents with anxiety and depressive disorders. Depress Anxiety. 2005;22(3):130–7.
16. Sawyer MG, Arney FM, Baghurst PA, et al. The mental health of young people in Australia: key findings from the child and adolescent component of the national survey of mental health and well-being. Aust N Z J Psychiatry. 2001;35(6):806–14.
17. Kataoka SH. Unmet need for mental health care among U.S. children: variation by ethnicity and insurance status. Am J Psychiatry. 2002;159(9):1548–1555.

18. Gulliver A, Griffiths KM, Christensen H. Perceived barriers and facilitators to mental health help-seeking in young people: a systematic review. BMC Psychiatry. 2010;10(1):113.
19. Ishikawa S, Okifuji A, Matsuoka H, Sakano Y. (2007). Cognitive Behaviour Therapy for Anxiety Disorders in Children and Adolescents: A Meta-Analysis. *Child and Adolescent Mental Health, 12*, 164-172.
20. James AC, James G, Cowdrey FA, Soler A, Choke A. (2013). Cognitive behavioural therapy for anxiety disorders in children and adolescents. *Cochrane database Syst Rev*. 6: CD004690.
21. Silverman WK, Pina AA, Viswesvaran C. (2008). Evidence-based psychosocial treatments for phobic and anxiety disorders in children and adolescents. *J Clin Child Adolesc Psychol, 37*, 105-130.
22. Reynolds S, Wilson C, Austin J, Hooper L. (2012). Effects of psychotherapy for anxiety in children and adolescents: A meta-analytic review. *Clinical Psychology Review, 32*, 251-262.
23. In-Albon T, Schneider S. Psychotherapy of childhood anxiety disorders: A meta-analysis. Psychother Psychosom 2007;76(1):15-24.
24. Cartwright-Hatton S, Roberts C, Chitsabesan P, Fothergill C, Harrington R. Systematic review of the efficacy of cognitive behaviour therapies for childhood and adolescent anxiety disorders. Br J Clin Psychol 2004 Nov;43(Pt 4):421-36.
25. DR1, den 30. september 2013, kl.21.30; *http://www.dr.dk/tv/se/tv-avisen/tv-avisen-788#!/05:20*, hentet den 09.10.13.
26. Kiropoulos, L. A., Klein, B., Austin, D. W., Gilson, K., Pier, C., Mitchell, J. & Ciechomski, L. (2008). Is internet-based CBT for panic disorder and agoraphobia as effective as face-to-face CBT? *Journal of Anxiety Disorders, 22*(8), 1273-1284.
27. Cunningham MJ, Wuthrich VM, Rapee RM, Lyneham HJ, Schniering CA, Hudson JL. The Cool Teens CD-ROM for anxiety disorders in adolescents : a pilot case series. Eur Child Adolesc Psychiatry. 2009;18(2):125–9.
28. Calear AL, Christensen H. Review of internet-based prevention and treatment programs for anxiety and depression in children and adolescents. Med J Aust. 2010;192(11 Suppl):S12–4.
29. Kendall, P. C., Khanna, M. S., Edson, A., Cummings, C., & Harris, M. S. (2011). Computers and psychosocial treatment for child anxiety: Recent advances and ongoing efforts. Depression and Anxiety, 28(1), 58-66. doi: http://dx.doi.org/10.1002/da.20757
30. Haug T, Nordgreen T, Ost LG, Havik OE. (2012). Self-help treatment of anxiety disorders: A meta-analysis and meta-regression of effects and potential moderators. *Clinical Psychology Review, 32*, 425-445.
31. Reger MA, Gahm GA. (2009). A meta-analysis of the effects of internet- and computer-based cognitive-behavioral treatments for anxiety. *J Clin Psychol, 65*, 53-75.
32. Andrews G, Cuijpers P, Craske MG, McEvoy P, Titov N. Computer therapy for the anxiety and depressive disorders is effective, acceptable and practical health care: a meta-analysis. PLoS One. 2010;5(10):e13196.
33. Haug T, Nordgreen T, Öst LG, Havik OE. Self-help treatment of anxiety disorders: a meta-analysis and meta-regression of effects and potential moderators. Clin Psychol Rev. 2012;32(5):425–45.
34. Andersson G, Cuijpers P, Carlbring P, Riper H, Hedman E. (In press). Internet-based vs. face-to-face cognitive behaviour therapy for psychiatric and somatic disorders: A systematic review and meta-analysis. World Psychiatry.
35. Khanna MS, Kendall PC. (2010). Computer-assisted cognitive behavioral therapy for child anxiety: Results of a randomized clinical trial. *J Consult Clin Psychol, 78*, 737-745.

36. March S, Spence SH, Donovan CL. (2009). The efficacy of an internet-based cognitive-behavioral therapy intervention for child anxiety disorders. *J Pediatr Psychol, 34*, 474-487.
37. Spence, S. H., Holmes, J. M., March, S., & Lipp, O. V. (2006). The feasibility and outcome of clinic plus Internet delivery of cognitive-behavior therapy for childhood anxiety. J Consult Clin Psychol, 74(3), 614-621. doi: http://dx.doi.org/10.1037/0022-006X.74.3.614
38. Spence SH, Donovan CL, March S, Gamble A, Anderson RE, Prosser S, Kenardy J. (2011). A randomized controlled trial of online versus clinic-based CBT for adolescent anxiety. *J Consult Clin Psychol, 79*, 629-642.
39. Wuthrich VM, Rapee RM, Cunningham MJ, Lyneham HJ, Hudson JL, Schniering CA. (2012). A Randomized Controlled Trial of the Cool Teens CD-ROM Computerized Program for Adolescent Anxiety. *Journal of the American Academy of Child and Adolescent Psychiatry, 51*, 261-270.
40. Tillfors M, Andersson G, Ekselius L, et al. A randomized trial of internet-delivered treatment for social anxiety disorder in high school students. Cogn Behav Ther. 2011;40(2):147–157.
41. Stallard, P., Richardson, T., Velleman, S., & Attwood, M. (2011). Computerized CBT (think, feel, do) for depression and anxiety in children and adolescents: Outcomes and feedback from a pilot randomized controlled trial. Behavioural and Cognitive Psychotherapy, 39(3), 273-284. doi: http://dx.doi.org/10.1017/S135246581000086X
42. Sethi S, Campbell AJ, Ellis LA. The use of computerized self-help packages to treat adolescent depression and anxiety. J Technol Hum Serv. 2010;28(3):144–160.
43. Stallard, P., Velleman, S. & Richardson, T. (2010). Computer use and attitudes towards computerized therapy amongst young people and parents attending child and adolescent mental health services. *Child and Adolescent Mental Health, 15*, 80-84.
44. James, A.M. (2007). Principles of youth participation in mental health services. *Medical Journal of Australia, 187*(suppl. 7), s57-s60.
45. Subrahmanyam, K. & Lin, G. (2007). Adolescents on the net: Internet use and well-being. *Adolescence, 42*, 659-677.
46. Livingstone, S., Haddon, L., Görzig, A., and Ólafsson, K. (2011). Risks and safety on the internet: The perspective of European children. Full Findings. LSE, London: EU Kids Online.
47. Arendt, K., Thastum, M., & Hougaard, E. (submitted). Efficacy of a Danish Version of the Cool Kids Program: A Randomized Wait-List Controlled Trial. Submitted to Behaviour Research and Therapy.
48. Gulliver, A., Griffiths, K. M., & Christensen, H. (2010). Perceived barriers and facilitators to mental health help-seeking in young people: A systematic review. BMC Psychiatry, 10. doi: http://dx.doi.org/10.1186/1471-244X-10-113
49. Kendall, P. C., Khanna, M. S., Edson, A., Cummings, C., & Harris, M. S. (2011). Computers and psychosocial treatment for child anxiety: Recent advances and ongoing efforts. Depression and Anxiety, 28(1), 58-66. doi: http://dx.doi.org/10.1002/da.20757
50. Richardson, T., Stallard, P., & Velleman, S. (2010). Computerised cognitive behavioural therapy for the prevention and treatment of depression and anxiety in children and adolescents: A systematic review. Clinical Child and Family Psychology Review, 13(3), 275-290. doi: http://dx.doi.org/10.1007/s10567-010-0069-9
51. Rickwood, D., & Bradford, S. (2012). The role of self-help in the treatment of mild anxiety disorders in young people: An evidence-based review. Psychology Research and Behavior Management, 5.

52. Rooksby, M., Elouafkaoui, P., Humphris, G., Clarkson, J., & Freeman, R. (2015). Internet-assisted delivery of cognitive behavioural therapy (CBT) for childhood anxiety: Systematic review and meta-analysis. Journal of Anxiety Disorders, 29(0), 83-92. doi: http://dx.doi.org/10.1016/j.janxdis.2014.11.006
53. Rapee, R., Lyneham, H., Schniering, C., Wuthrich, V., Abbott, M., Hudson, J. & Wignall, A. (2006). *Cool Kids "Chilled" Adolescent Anxiety Program.* Centre for Emotional Health, Macquarie University, Sydney.
54. Rapee RM, Lyneham HJ, Schniering CA, Wuthrich V, Abbott MJ, Hudson JL, Wignall A. (2010). *Cool Kids angstprogram for børn. Terapeutens manual.* Psykologisk Institut, Aarhus Universitet.
55. DSM-IV-TR. (2000). *Diagnostic and Statistical Manual of Mental Disorders, 4th ed. Text Revision.* Arlington, VA.: American Psychiatric Association.
56. Albano AM, Silverman WK. (1996). *Anxiety Disorders Interview Scedule for DSM - IV. Child version*. Oxford, Oxford University Press.
57. Lyneham HJ, Rapee RM. (2005). Agreement between telephone and in-person delivery of a structured interview for anxiety disorders in children. *Journal of the American Academy of Child and Adolescent Psychiatry, 44*, 274-282.
58. Cobham, V. E., Dadds, M. R., & Spence, S. H. (1999). Anxious children and their parents: What do they expect? Journal of Clinical Child Psychology, 28, 220–231. doi:10.1207/s15374424jccp2802_9
59. Arendt, K., Hougaard, E., & Thastum, M. (2014). Psychometric properties of the child and parent versions of Spence Children's Anxiety Scale in a Danish community and clinical sample. Journal of Anxiety Disorders, 28(8), 947-956. doi: http://dx.doi.org/10.1016/j.janxdis.2014.09.021
60. Nauta, M. H., Scholing, A., Rapee, R. M., Abbott, M., Spence, S. H., & Waters, A. (2004). A parent-report measure of children's anxiety: Psychometric properties and comparison with child-report in a clinic and normal sample. Behaviour Research and Therapy, 42(7), 813-839. doi: http://dx.doi.org/10.1016/S0005-7967(03)00200-6
61. Lyneham, H. J., Sburlati, E. S., Abbott, M. J., Rapee, R. M., Hudson, J. L., Tolin, D. F., & Carlson, S. E. (2013). Psychometric properties of the Child Anxiety Life Interference Scale (CALIS). Journal of Anxiety Disorders, 27(7), 711-719. doi: http://dx.doi.org/10.1016/j.janxdis.2013.09.008
62. Muris, P. (2001). A brief questionnaire for measuring self-efficacy in youths. Journal of Psychopathology and Behavioral Assessment, 23(3), 145-149. doi: http://dx.doi.org/10.1023/A:1010961119608
63. Muris, P. (2002). Relationships between self-efficacy and symptoms of anxiety disorders and depression in a normal adolescent sample. Personality and Individual Differences, 32(2), 337-348. doi: http://dx.doi.org/10.1016/S0191-8869(01)00027-7
64. Angold, A., Costello, E. J., Messer, S. C., & Pickles, A. (1995). Development of a short questionnaire for use in epidemiological studies of depression in children and adolescents. International Journal of Methods in Psychiatric Research, 5(4), 237-249.
65. Goodman, R., & Scott, S. (1999). Comparing the Strengths and Difficulties Questionnaire and the Child Behavior Checklist: Is small beautiful? J Abnorm Child Psychol, 27(1), 17-24. doi: http://dx.doi.org/10.1023/A:1022658222914
66. Niclasen, J., Teasdale, T. W., Andersen, A.-M. N., Skovgaard, A. M., Elberling, H., & Obel, C. (2012). Psychometric properties of the Danish Strength and Difficulties Questionnaire: The SDQ assessed for more than 70,000 raters in four different cohorts. PLoS One, 7(2). doi: http://dx.doi.org/10.1371/journal.pone.0032025

67. Horvath, A. O. (1981). An exploratory study of the working alliance: Its measurement and relationship to therapy outcome. Retrieved from http://search.proquest.com/docview/616700966? accountid=14468
68. Tracey, T. J., & Kokotovic, A. M. (1989). Factor structure of the Working Alliance Inventory. Psychological Assessment: A Journal of Consulting and Clinical Psychology, 1(3), 207-210. doi: http://dx.doi.org/10.1037/1040-3590.1.3.207

Bilag 1: Projektets effektmål

- ADIS-c/p pr. telefon (præ, post, follow-up)
- Demografispørgsmål (barnets alder og køn, familiekonstellation, privat/folkeskole, barnets gennemsnitlige antal skolefraværsdage, tidligere søgt hjælp til barnets angst, eventuel medicinering af barnet, forælderens alder, ægteskabelige status, antal børn, skoleuddannelse, afsluttende klassetrin, uddannelsesniveau, beskæftigelse, indkomst) (præ) F
- Spence Child Anxiety Scale (SCAS; 2,59,60) (præ, post, follow-up) U/F
- Children's Anxiety Life Inference Scale (CALIS; 61) (præ, post, follow-up) U/F
- Self-Efficacy Questionnaire for Children (SEQ-c; 62, 63) (præ, post) U
- Short Moods and Feelings Questionnaire (SMFQ-c/p; 64) (præ, post) U/F
- Quality of life (WHO5's questionnaire, 5 items) (præ, post, follow-up) U
- Strength and Difficulties Questionnaire for Youth (SDQ; 65, 66) (præ, post) U/F
- Working Alliance Inventory – Short form (WAI-S; 67; 68) (efter 4. og 8. uge, post) U
- Experience of Service Questionnaire (ESQ; Commission for Health Improvement, 2002) (post) U/F
- Adherence, dvs. grad af programgennemførsel, antal gange (evt. samlet tid) logget ind i programmet (monitoreres i programmets back end) (post) U

Yderligere spørgsmål

- Hvordan har du det med at skulle i behandling? (præ) U
- Hvordan har du det med, at din behandling kommer til at foregå over internettet? (præ) U
- Hvor fortrolig er du med brugen af computer og internet? (præ) U
  Slet ikke Lidt Rimelig Meget
- Hvor langt er du kommet i programmet? (post) U
- Hvor meget tid har du i gennemsnit pr. uge brugt på at arbejde med modulerne i det online program? (midt, post) U
  Ingen tid 10-30 min 30-60 min. 1-2 timer 2-5 timer 5-10 timer Mere end 10 timer
- Hvor meget tid har du i gennemsnit pr. uge brugt på øvelser ud over tiden ved computeren? (midt, post) U
  Ingen tid 10-30 min 30-60 min. 1-2 timer 2-5 timer 5-10 timer Mere end 10 timer
- Hvor meget tid har du i gennemsnit pr. uge brugt på at hjælpe dit barn med gennemførelsen af programmet? (midt, post) F
  Ingen tid 10-30 min 30-60 min. 1-2 timer 2-5 timer 5-10 timer Mere end 10 timer

Præ = før behandlingsstart
Midt = 7. uge
Post = 15 uger efter behandlingsstart
Follow-up = 3 mdr. efter behandlingsafslutning
U = udfyldes af den unge
F = udfyldes af begge forældre
